# Novel Insights on Ferroptosis Modulation as Potential Strategy for Cancer Treatment: When Nature Kills

**DOI:** 10.1089/ars.2022.0179

**Published:** 2024-01-17

**Authors:** Valeria Consoli, Antonino Nicolò Fallica, Valeria Sorrenti, Valeria Pittalà, Luca Vanella

**Affiliations:** ^1^ Department of Drug and Health Sciences, University of Catania, Catania, Italy.; ^2^ Department of Drug and Health Sciences, CERNUT—Research Centre on Nutraceuticals and Health Products, University of Catania, Catania, Italy.

**Keywords:** ferroptosis, natural compounds, ferroptosis inducers, cancer, anticancer strategies, phytochemicals, oxidative stress

## Abstract

**Significance:**

The multifactorial nature of the mechanisms implicated in cancer development still represents a major issue for the success of established antitumor therapies. The discovery of ferroptosis, a novel form of programmed cell death distinct from apoptosis, along with the identification of the molecular pathways activated during its execution, has led to the uncovering of novel molecules characterized by ferroptosis-inducing properties.

**Recent advances:**

As of today, the ferroptosis-inducing properties of compounds derived from natural sources have been investigated and interesting findings have been reported both *in vitro* and *in vivo*.

**Critical Issues:**

Despite the efforts made so far, only a limited number of synthetic compounds have been identified as ferroptosis inducers, and their utilization is still limited to basic research. In this review, we analyzed the most important biochemical pathways involved in ferroptosis execution, with particular attention to the newest literature findings on canonical and non-canonical hallmarks, together with mechanisms of action of natural compounds identified as novel ferroptosis inducers. Compounds have been classified based on their chemical structure, and modulation of ferroptosis-related biochemical pathways has been reported.

**Future Directions:**

The outcomes herein collected represent a fascinating starting point from which to take hints for future drug discovery studies aimed at identifying ferroptosis-inducing natural compounds for anticancer therapies. *Antioxid. Redox Signal.* 40, 40–85.

**Figure:**
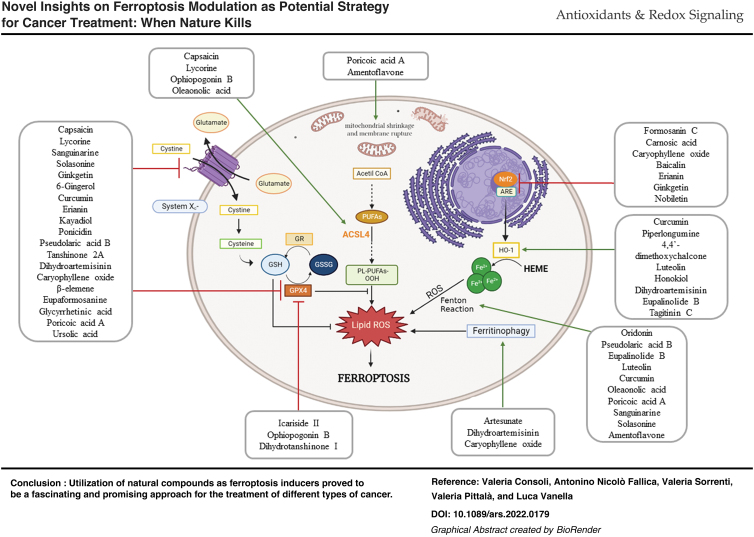
Color images are available online.

## Table of Contents

I. Introduction  43
II. Ferroptosis and Tumor Microenvironment  44
III. Ferroptosis Key Players  45
  A. Iron homeostasis  45
  B. Lipid metabolism  46
  C. Antioxidant systems  48
     1. Glutamate-cysteine ligase  48
     2. Multidrug resistance protein 1  48
     3. Reduced nicotinamide adenine dinucleotide phosphate  50
     4. Ferroptosis suppressor protein 1  50
     5. GCH1-BH4 axis  50
     6. Microsomal glutathione S-transferase 1  51
  D. Tumor suppressor p53  51
  E. Non-coding RNAs  52
IV. Ferroptosis in Cancer  52
  A. Lung cancer  52
  B. Colorectal cancer  54
  C. Hepatic cancer  55
  D. Gastric cancer  55
  E. Breast cancer  55
V. Natural Compounds as Ferroptosis Inducers  56
  A. Alkaloids  56
     1. Capsaicin  56
      i. *In vitro*  56
   2. Lycorine  56
      i. *In vitro*  56
   3. Piperlongumine  56
      i. *In vitro*  56
   4. Sanguinarine  56
      i. *In vitro*  56
   5. Solasonine  57
      i. *In vitro*  57
      ii. *In vivo*  57
  B. Flavonoids  57
   1. 4,4′-Dimethoxychalcone  57
      i. *In vitro*  57
   2. Amentoflavone  57
      i. *In vitro* and *in vivo*  57
   3. Baicalin  57
      i. *In vitro* and *in vivo*  57
   4. Ginkgetin  58
      i. *In vitro* and *in vivo*  58
   5. Icariside II and luteolin  58
      i. *In vitro* and *in vivo*  58
   6. Nobiletin  58
      i. *In vitro*  58
  C. Phenols and polyphenols  58
   1. 6-Gingerol  58
      i. *In vitro*  58
   2. Curcumin  59
      i. *In vitro*  59
      ii. *In vivo*  59
   3. Erianin  59
      i. *In vitro* and *in vivo*  59
   4. Honokiol  59
      i. *In vitro*  59
  D. Saponins  59
   1. Formosanin C  60
      i. *In vitro*  60
   2. Ophiopogonin B  60
      i. *In vitro* and *in vivo*  60
  E. Terpenes and terpenoids  60
   1. Carnosic acid  60
      i. *In vitro*  60
   2. Dihydrotanshinone I and tanshinone 2A  60
      i. *In vitro*  60
      ii. *In vivo*  61
   3. Kayadiol  61
      i. *In vitro*  61
   4. Oridonin and ponicidin  61
      i. *In vitro*  61
   5. Pseudolaric acid B  61
      i. *In vitro* and *in vivo*  61
   6. Artesunate and dihydroartemisinin  61
      i. *In vitro* and *in vivo*  61
   7. Caryophyllene oxide  62
      i. *In vitro*  62
   8. β-Elemene  62
      i. *In vitro* and *in vivo*  62
   9. Eupaformosanine  62
      i. *In vitro* and *in vivo*  62
   10. Eupalinolide B and tagitinin C  63
      i. *In vitro* and *in vivo*  63
      ii. *In vitro*  63
   11. Glycyrrhetinic acid and oleanolic acid  63
      i. *In vitro*  63
      ii. *In vitro* and *in vivo*  63
   12. Poricoic acid A  63
      i. *In vitro* and *in vivo*  63
   13. Ursolic acid  63
      i. *In vitro* and *in vivo*  63
VI. Current Ferroptosis Limitations and Advances Beyond *In Vitro* Research  64
VII. Conclusions and Future Perspectives  64

## I. Introduction

Cell death is an inevitable process that marks the fate of all living creatures, either in a physiological or pathological manner. Apoptosis can be considered the most well characterized and prevalent form of controlled cell death, while uncontrolled cell death results in necrosis. However, other types of controlled cell death have been discovered and characterized such as autophagy, necroptosis, pyroptosis and ferroptosis (D'Arcy et al., 2019; Yan et al., 2020).

Over the years, several articles have reported about lipid peroxidation (LPO) and glutathione (GSH) depletion-dependent cell death reflecting different features that are today related to ferroptosis (Barrera et al., 2008; Efferth et al., 1996; Kinsey et al., 2008; Linden et al., 2008), even though ferroptosis was first described by Dixon et al. in 2012 using RAS-selective lethal (RSL) small molecules, such as erastin and RAS-selective lethal molecule 3 (RSL3), as triggers of the process. In 2018, the Nomenclature Committee on Cell Death officially defined ferroptosis as a form of regulated cell death (Galluzzi et al., 2018).

Morphologically, ferroptosis displays peculiar features as reduced mitochondrial volume and mitochondrial shrinkage, increased membrane density, and reduction or disappearance of mitochondrial cristae, together with a rounded morphology of the cell undergoing ferroptotic death; nevertheless, the cell membrane remains intact, nucleus size does not show alterations, and there is no concentration of chromatin, clearly differentiating it from the apoptosis mechanism (Dixon et al., 2012; Mou et al., 2019). Ferroptosis execution is mainly characterized by an extensive iron-dependent reactive oxygen species (ROS) production and subsequent peroxidation of polyunsaturated fatty acids (PUFAs)-containing phospholipids (PLs), as Fe^2+^ oxidizes lipids through the Fenton reaction.

**FIG. 1.**
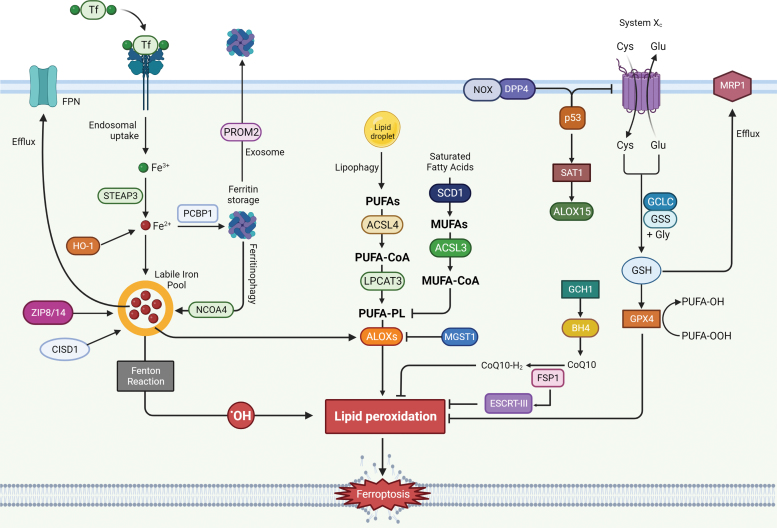
**Schematic representation of the main signaling pathways involved in ferroptosis.** ACSL3, acyl-CoA synthetase long chain family member 3; ACSL4, acyl-CoA synthetase long chain family member 4; ALOX, lipoxygenase; BH4, tetrahydrobiopterin; CISD1, CDGSH Iron Sulfur Domain 1; DPP4, Dipeptidyl-peptidase-4; ESCRT-III, endosomal sorting complex required for transport-III; FPN, ferroportin-1; FSP1, ferroptosis suppressor protein 1; GCH1, GTP cyclohydrolase-1; GCLC, glutamate-cysteine ligase catalytic subunit; GPX4, glutathione peroxidase 4; GR, GSH reductase; GSH, glutathione; GSS, GSH synthetase; GSSG, GSH disulfide; HO-1, heme oxygenase-1; LPCAT3, lysophosphatidylcholine acyltransferase 3; MRP1, multidrug resistance protein 1; MUFA, monounsaturated fatty acids; MUFA-CoA, MUFA-acyl-CoA esters; NADPH, reduced nicotinamide adenine dinucleotide phosphate; NCOA4, nuclear receptor coactivator 4; NOX, NADPH oxidase; p53, tumor protein p53; PCBP1, poly (rC)–binding protein 1; PROM2, prominin 2; PUFA, polyunsaturated fatty acid; PUFA-CoA, PUFA-acyl-CoA esters; PUFA-PL, phospholipid PUFA; SAT1, spermidine/spermine N1-acetyltransferase 1; SCD1, stearoyl CoA desaturase 1; STEAP3, six-transmembrane epithelial antigen of the prostate 3; ZIP, ZRT/IRT-like protein. Color images are available online.

In this context, intracellular GSH depletion and decreased activity of glutathione peroxidase 4 (GPX4) sustain the ferroptotic process, unravelling the crucial interplay between lipid metabolism, antioxidant defenses, and iron homeostasis (Fig. 1) (Mao et al., 2021; Sun et al., 2020). Targets of ferroptosis inducers, for instance erastin and RSL3, are specific inhibitors of the cystine/glutamate antiporter system xc^−^ and GPX4, respectively. Further, ACSL4 (acyl-CoA synthetase long chain family member 4) dictates sensitivity to ferroptosis and, usually, its expression is upregulated in some types of cancer rather than in healthy cells, determining a potential selectivity for cancer cells (Zhao et al., 2020b).

In light of the unique features observed in ferroptotic cell death, it could be possible to establish new potential anticancer treatments that may overcome multidrug-resistance phenomena related to the inactivation of specific apoptotic signaling pathways or altered targets encountered in resistant cancer cells. Considering this, in recent years, research is extensively focusing on the elucidation of biochemical pathways responsible for ferroptosis induction, on their interplay, and on the search for molecular tools exploitable for such purposes.

**FIG. 2.**
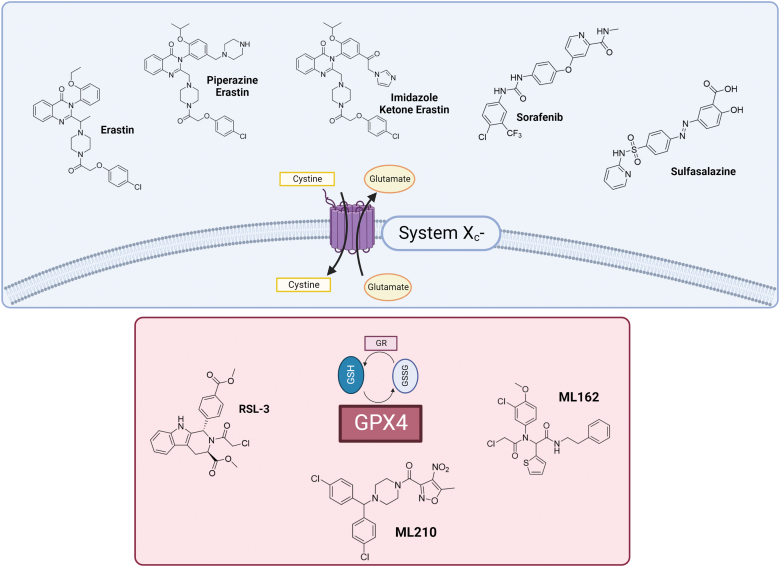
**Representation of the principal xc^‒^ system and GPX4 inhibitors.** Color images are available online.

Remarkably, the recognition of GPX4 and xc^‒^ system as pivotal actors harmonizing the ferroptotic process could be of particular usefulness for the design of new molecular entities aiming at inhibiting their biological activity and promoting cell death. Currently, the most relevant compounds used and studied as ferroptosis inducers comprise synthetic inhibitors of xc^‒^ system and GPX4 (Fig. 2). However, except for erastin and its derivatives, sulfasalazine (SAS), RSL3, ML162, and ML210, poor results have been achieved hitherto for the obtainment of novel inducers (Liang et al., 2019).

Nature represents an inexhaustible reservoir from which to draw for the discovery of new compounds with potential biological activity. Nowadays, the discovery of new therapeutic compounds from natural sources is once more gaining great attention from the scientific community, since the use of novel drug discovery approaches such as combinatorial chemistry led to the identification of only three compounds that received approval from the FDA (U.S. Food and Drug Administration) since 1981 (Newman and Cragg, 2020). On the other hand, among the approved small molecules reported from 1981 to 2019, the 32% were represented by natural or natural-derived compounds, and from this total amount 53.3% of molecules are labeled as anticancer agents.

In addition, the evolution of modern approaches for natural products drug discovery could speed up the identification of novel molecules to be used for potential pharmacological therapies (Najmi et al., 2022; Thomford et al., 2018). The great advantages regarding the utilization of phytochemical compounds reside in their easier provision, potential lower toxicity and mostly in their variegated structural diversity, which can allow the identification of common scaffolds responsible for a precise therapeutic effect.

In light of these considerations, we herein describe the key features of ferroptosis, classic and non-canonical pathways involved in its execution, and the most promising natural compounds acting as positive ferroptosis effectors together with their mechanism of action in the tested tumor cell lines for the establishment of potential anticancer therapeutic regimens.

## II. Ferroptosis and Tumor Microenvironment

Several lines of evidence have shown ferroptosis regulatory role in the occurrence and development of many pathological conditions, including tumor suppressing pathways, making it a hotspot in the field of antineoplastic therapy (Jiang et al., 2015; Liang et al., 2019).

Apart from cancer cells, tumor microenvironment also comprises immune cells, including T cells, macrophages, and myeloid-derived suppressor cells (MDSCs), which are subjected to similar growth signals and metabolic properties as cancer cells. Cancer cells can recruit tumor-associated macrophages (TAMs) massively, whose predominantly pro-tumor M2 phenotype leads to immunosuppression. Recent studies concerning tumor therapies targeting macrophages are mainly aimed at the repolarization, changing TAM M2 phenotype to M1 phenotype (Yang et al., 2022d). The MDSCs in the tumor microenvironment have potent immunosuppressive capacity exhibiting resistance to ferroptosis.

Indeed, ferroptosis promotion of MDSC would be a promising approach for improving the tumor immunosuppressive microenvironment (Dang et al., 2022). Simultaneously inhibiting ferroptosis of anti-tumor immune cells and promoting ferroptosis of immunosuppressive immune cells could enhance the benefits of cancer immunotherapy; moreover, the immunogenic effect of ferroptosis activation could represent a promising tool for anticancer treatment (Friedmann Angeli et al., 2019; Tang et al., 2020).

Data reported by Wang et al. show ferroptosis involvement in T cell-mediated cancer immunity. Besides apoptosis and senescence, ferroptosis activation in tumor cells seems to be a previously unappreciated mechanism for CD8+ T cell-mediated tumor clearance *in vivo*. Interferon gamma (IFNγ) released from CD8+ T cells downregulates the expression of Solute Carrier Family 3 Member 2 (SLC3A2) and solute carrier family 7 member 11 (SLC7A11), resulting in impairment of cystine uptake and enhancement of lipid peroxidation, ultimately triggering ferroptosis.

Moreover, cystine depletion combined with immune checkpoint inhibitors (ICIs) synergistically enhanced T cell-mediated anti-tumor immunity and ferroptosis (Wang et al., 2019c). However, it has been shown that immunosuppressive polymorphonuclear myeloid-derived suppressor cells (PMN-MDSCs) play a crucial role in tumor microenvironment and lead to immunotherapy resistance with poor clinical outcome in cancer patients.

Moreover, spontaneous ferroptotic cell death was observed in PMN-MDSCs, which promotes tumor growth through immuno-restrictive activity. In some models, ferroptosis immunosuppressive effect in PMN-MDSCs can outweigh its tumor-killing effect toward cancer cells. Thus, specific cell targeting seems to be necessary to exploit both induction and inhibition of ferroptosis, which can lead to the same therapeutic outcome, tumor cell death, and immunosuppression bust (Du et al., 2023a; Kim et al., 2022).

## III. Ferroptosis Key Players

In the following paragraphs, canonical and noncanonical ferroptosis as key players will be discussed.

### A. Iron homeostasis

Iron plays a pivotal role as the fundamental component of several enzymes involved in processes such as angiogenesis, cell proliferation, DNA synthesis, and metastasization. Nevertheless, iron acts as a redox-active reagent and promotes the production of free radicals and other strongly oxidizing species *via* Fenton reaction, potentially causing a wide range of biological damage (Andrews and Schmidt, 2007; Winterbourn, 1995). Most organisms have to find the challenging balance of acquiring the appropriate amount of iron for essential biological processes while avoiding its potential toxicity.

**FIG. 3.**
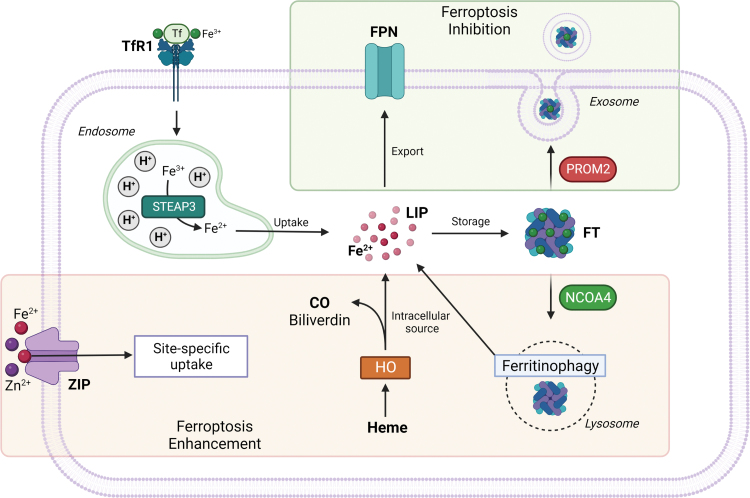
**Schematic representation of cellular iron homeostasis and its impact on the ferroptotic process.** CO, carbon monoxide; FT, ferritin; HO, heme oxygenase; TfR1, transferrin receptor 1. Color images are available online.

Ceruloplasmin is responsible for catalyzing oxidation of Fe^2+^ to Fe^3+^, which is then bound to transferrin (TF) to form the complex TF-Fe3+. TF-bound iron interacts with membrane protein TF receptor1 (TfR1), whose accumulation on cell surface has recently been identified as a ferroptotic feature (Feng et al., 2020). Once Fe^3+^ gets inside the cell by endocytosis, it can be reduced to Fe^2+^ by the six-transmembrane epithelial antigen of the prostate 3 (STEAP3) and subsequently build up a labile iron pool (LIP), or be stored by ferritin (FT), a protein complex consisting of ferritin light chain (FTL) and ferritin heavy chain 1 (FTH1) (Fig. 3) (Arosio et al., 2010; Bogdan et al., 2016).

Heme oxygenase 1 (HO-1), as the rate limiting enzyme that catalyzes the degradation of heme to ferrous iron, biliverdin, and carbon monoxide, can provide a source of reactive iron in cells; thus, its overexpression and particularly its overactivation can promote ferroptosis (Fig. 3) (Chang et al., 2018; Consoli et al., 2022; Hassannia et al., 2018). Consistently with these findings, it was shown that silencing or enzymatic inhibition of HO-1 can reverse erastin, withaferin A, and BAY 11-7085-induced ferroptosis (Kwon et al., 2015).

However, HO-1 can also exert cytoprotective functions (Sun et al., 2016b), depending on the extent of its activation. HO-1 protective effects are associated with its antioxidant activity, whereas the augmented generation of ferrous iron promotes the accumulation of free radicals *via* the Fenton reaction beyond the buffering capacity of the cell, ultimately leading to cytotoxic consequences. Therefore, excessive or moderate HO-1 upregulation can exert, respectively, detrimental or beneficial effects also depending on cellular redox balance (Consoli et al., 2021; Gorrini et al., 2013).

Increased iron uptake and reduced iron storage may contribute to iron overload during ferroptosis. Free iron, in both Fe^2+^ and Fe^3+^ form, is essential for ferroptosis initiation as it is responsible for lipid peroxidation (Minotti and Aust, 1987); therefore, iron chelators are able to inhibit ferroptotic cell death by reducing free iron availability, resulting in lower lipid peroxides cellular content (Xu et al., 2019). Human poly (rC)–binding protein 1 (PCBP1) has been reported to be responsible for facilitating iron loading into FT *in vitro*. PCBP1 is a member of a family of four homologous widely expressed and highly conserved among mammals that can also bind RNA.

FT first binds iron atoms in the ferrous form (Fe^2+^), then the ferroxidase center of H-FT provides for oxidation to ferric iron, located in the interior of the FT cavity (Philpott and Ryu, 2014). Work by Shi et al. (2008) reported that PCBP1 downregulation *in vitro* led to reduced FT iron incorporation and increased LIP, whereas PCBP1 overexpression enhanced FT iron incorporation efficiency. Silencing of iron-responsive element binding protein 2 (IREB2), a master transcription factor of iron metabolism, significantly limits erastin-induced ferroptosis (Dixon et al., 2012).

In recent years, studies have focused their attention on ZRT/IRT-like protein (ZIP) family, which counts 14 members and has been associated with transmembrane zinc transport, revealing a crucial role for two ZIP family members in iron transport: ZIP8 and ZIP14. Both were found to mediate the non-transferrin-bound iron (NTBI) uptake and transport across the cell membrane and seem to be correlated to iron overload diseases (Fig. 3) (Pinilla-Tenas et al., 2011; Wang et al., 2012).

Wu et al. revealed a new role of divalent metal transporter ZIP14 as potentially responsible for the initiation of ferroptosis in both *in vitro* and *in vivo* models of diabetic nephropathy. They observed ZIP14 up-regulation and Fe^2+^ increased levels associated with reduced expression of GPX4 and low levels of GSH, whereas malondialdehyde (MDA) levels were increased (Wu et al., 2022) consistently with ferroptosis onset.

On the other hand, results from a study on cadmium-associated/testis-related ferroptosis seems to exclude the contribution of increased importation of extracellular iron through ZIP8. Indeed, ferroptosis in the testes was mainly due to reduction of iron export and storage, together with inhibition of SLC7A11 (Xiong et al., 2022). Taken together, these observations indicate site-specific actions of ZIP transporters, which need more in-depth investigation to be correlated with ferroptosis onset.

It was observed that the inhibition of iron export *via* either the solute carrier family 40 member 1 (SLC40A1, also known as ferroportin-1 or FPN) (Li et al., 2018a; Ma et al., 2016) autophagic degradation or through the blockage of prominin 2 (PROM2, a transmembrane glycoprotein) and lipocalin 2 (LCN2, a siderophore-binding protein) (Brown et al., 2019; Liu et al., 2021a) seem to enhance ferroptosis susceptibility under various circumstances (Fig. 3).

Another ferroptosis promoting factor is represented by the increase of the mitochondrial iron uptake following mitochondrial iron exporter CDGSH Iron Sulfur Domain 1 (CISD1), an iron–sulfur cluster protein also known as mitoNEET, or CDGSH Iron Sulfur Domain 2 (CISD2) inhibition (Kim et al., 2018; Yuan et al., 2016), concurrently with the reduction of iron used for iron–sulfur cluster biosynthesis (Alvarez et al., 2017). Recently, nuclear receptor coactivator 4 (NCOA4) has been identified as the protein responsible for mediating ferritinophagy through FT delivery into lysosomes. In conditions of starvation or iron depletion, NCOA4 was found to bind FTH1 to target the iron-binding FT complex (Fig. 3) (Dowdle et al., 2014; Mancias et al., 2014).

Ferritinophagy modulation has paved the way to some interesting opportunities for innovative cancer treatment approaches. For instance, as discussed in the Breast Cancer section, the antimalarial drug artesunate was observed to accumulate within lysosomes of cancer cells and enhance lysosomal activity, which, in turn, accelerated autophagic degradation of FT leading to ferroptotic cell death. Artesunate was also found to synergistically act with sorafenib to trigger ferroptosis in hepatocellular carcinoma (HCC) (Bogdan et al., 2016; Li et al., 2021; Yang et al., 2014a).

Several studies have reported artesunate ability to induce ferroptosis in cancer not only through ferritinophagy activation but also *via* GSH depletion (Chen et al., 2020a; Eling et al., 2015; Roh et al., 2017; Tang et al., 2018). Hayashima et al. (2021) reported the correlation between GSH cellular content and ferritinophagy in glioblastoma. It was observed that cystine deprivation is able to induce NCOA4-mediated ferritinophagy to initiate ferroptosis in T98G and A172 glioblastoma cells. Inhibition of NCOA4 reversed ferroptotic cell death, suggesting that cystine deprivation-induced ferroptosis requires both GSH depletion and intracellular iron accumulation to be activated.

Another important finding was obtained by Yin et al. (2022) whose work highlighted the correlation between GPX4 inhibition and NCOA4-mediated ferritinophagy activation in tetrandrine citrate (TetC)-induced ferroptotic cell death in breast cancer (BC) cells. Therefore, reduction of iron storage by ferritinophagy-mediated FT degradation ultimately promotes ferroptosis execution (Hou et al., 2016).

### B. Lipid metabolism

Lipid peroxidation, with a particular attention for PUFAs, has been involved in the etiology of several pathological conditions, including cancer (Gaschler and Stockwell, 2017; Sun et al., 2019). PUFAs are generally represented by arachidonic acid (AA), linoleic acid, and docosahexaenoic acids. AA exerts important cellular functions as membrane integrity maintenance and synthesis of many bioactive mediators as leukotrienes (LTs), prostaglandins, thromboxane A2, epoxyeicosatrienoic acid, and endocannabinoids (Li et al., 2022e; Werz et al., 2003).

**FIG. 4.**
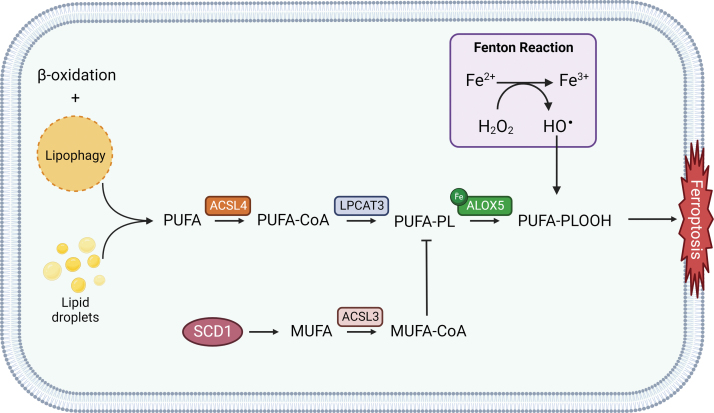
**Schematic representation of lipid peroxidation pathways involved in ferroptosis onset.** PUFA-PLOOH, PUFA-phospholipid hydroperoxide. Color images are available online.

PUFA oxidation can occur by either non-enzymatic free radical chain reaction or enzyme catalysis (Kagan et al., 2017). PUFAs can be considered a double-edged sword due to the cytotoxic effect following peroxidation. Those responsible for their integration into membranes are ACSL4 and lysophosphatidylcholine acyltransferase 3 (LPCAT3) (Fig. 4) (Dixon et al., 2015). Lipidomic analysis from Doll et al. (2017) studies have demonstrated ACSL4 ability to shape the lipid profile required for ferroptosis execution.

Moreover, ACSL4 has also emerged as a predictive factor for ferroptosis sensitivity. This study also highlighted an important functional interplay between GPX4 and ACSL4, as these genes double knockout surprisingly displayed a maintenance of cell viability for a sufficiently long period of time. Despite ACSL4 predominant role in ferroptosis susceptibility, it was observed that ACSL4 function is mandatory only for erastin and GPX4 inhibitors-induced cell death but not for tumor protein p53 (p53)-mediated ferroptosis. Indeed, studies by Chu et al. (2019) using ACSL4-null cells confirmed that p53-mediated ferroptosis is disentangled from ACSL4 activity, whereas erastin or RSL3 treatment proved to be ineffective (Kagan et al., 2017).

Interestingly, even if p53 and erastin have the same target, which is transporter SLC7A11, they activate very different mechanisms, as it can be deduced by the fact that p53 activity, unlike erastin, is lipoxygenase 12 (ALOX12) dependent.

Cyclooxygenases, cytochrome p450s, and ALOXs are the main enzymatic systems deputized to metabolize AA. Several ALOXes can directly oxidize PLs to generate hydroperoxy-eicosatetraenoic acid–phosphatidylethanolamines (HpETE–PEs) that serve as substrates for GPX4 to form the reduced products hydroxy-eicosatetraenoic acid–phosphatidylethanolamines (HETE–PEs) (Tyurina et al., 2019).

ALOX family, counting six functional subtypes in humans (lipoxygenase 3 [ALOXE3], ALOX5, ALOX12, ALOX12B, ALOX15, and ALOX15B), gives the principal contribution in lipid peroxides generation (Li et al., 2018b; Singh and Rao, 2019). In particular, ALOX5 serves as a rate-limiting enzyme responsible for the biosynthesis of LTs, which are the major mediators of inflammation, finally causing cancers and other pathological conditions. Further, ALOX12 and ALOX15 isoforms have been observed to be implicated as key regulators in ferroptosis.

ALOX12 has been observed to be crucial but not for GPX4 and ACSL4-mediated ferroptosis. In addition, its inactivation abrogates ROS/p53-mediated ferroptosis and suppresses p53 tumor suppressive function *in vivo*. Thus, a role of ALOX12 was suggested in p53-dependent activation of ferroptosis process. A study by Chu et al. (2019) tried to address the existing link between ALOX family and p53-mediated cell death, discovering ALOX12 pivotal role in ROS-mediated activation of p53 tumor suppressive protein.

Moreover, they reported the strict correlation of ALOX12 with SLC7A11 transporter, which is a fundamental player in ferroptosis and also a direct p53 target (Chu et al., 2019). ALOX15 is responsible for direct oxidation of AA-PE (arachidonic acid-phosphatidylethanolamine) and AdA (adrenic acid)-phosphatidylethanolamine (PE) into lipid hydroperoxides, which serve as pro-ferroptotic signals (Doll et al. 2017).

Li et al. (2018b) showed that ALOX15 isoforms (1,2) can be regulated by phosphatidylethanolamine-binding protein 1 (PEBP1), a scaffold protein inhibitor of protein kinase cascades. Indeed, they observed a shift in ALOX15 specificity following PEBP1/15-LOX complexes formation, resulting in hydroperoxy-eicosatetraenoic acid-phosphatidylethanolamines production from PUFA-PE, which exert pro-ferroptotic activity (Li et al., 2018b; Zhao et al., 2020a).

Moreover, liproxstatin-1 (Lip-1) was observed to be able to inhibit ALOX15 enzymatic activity and abolish the production of oxygenated PE *in vivo*, remarking on ALOXs contribution to ferroptosis (Kagan et al., 2017).

ALOX5 is a crucial enzyme that mediates lipid peroxidation by producing lipid peroxides, and as an iron-containing enzyme it has been strictly correlated to ferroptosis (Yang et al., 2016). Indeed, several lines of evidence have proposed ALOX5 as a target for ferroptosis (Liu et al., 2015; Shah et al., 2018; Sun et al., 2019; Xu and Chen, 2021). Inhibition of ALOX5 operated by the microsomal glutathione S-transferase 1 (MGST1) was observed to be consistent in human pancreatic ductal adenocarcinoma (PDAC) cell lines, remarking on ALOX5 role as a positive regulator of ferroptosis (Kuang et al., 2021).

Recently, stearoyl CoA desaturase 1 (SCD1) (Fig. 4), an enzyme that catalyzes the rate-limiting step in monounsaturated fatty-acid synthesis, has been associated with ferroptosis resistance in a number of cancers and correlated with a worse prognosis (Liu et al., 2022b; Luis et al., 2021; Tesfay et al., 2019; Ye et al., 2021). SCD1 was observed to be implicated in cell growth, survival, and cancer progression (Kikuchi and Tsukamoto, 2020); thus, SCD1 inhibition seems to be an appealing novel approach for enhancing ferroptosis sensitivity and triggering cellular death.

Desaturases such as SCD1 and fatty acid desaturase 2 (FADS2) are able to inhibit ferroptosis in BC cells (Li et al., 2022e). Knockdown of SCD1 enhanced the sensitivity of BC cells to ferroptosis inducers such as RSL3 and erastin. Indeed, SCD1 inhibition determined a reduction of coenzyme Q10 (CoQ10) and unsaturated fatty acyl chains in membrane PLs, whereas long-chain saturated ceramides were increased. Increased activity of SCD1 was observed to be related to ferroptosis inhibition as it likely increases the monounsaturated fatty acid (MUFA) production, which inhibits the accumulation of membrane lipid ROS and displaces PUFAs from their cellular location. Thus, SCD1 activity is responsible for the suppression of PUFAs peroxidation and subsequent ferroptosis activation (Magtanong et al., 2019).

A combination of ferroptosis inducers and SCD1 inhibitors seems to synergistically reduce cancer cells proliferation, providing a new potential treatment strategy.

Moreover, inhibition of β-oxidation together with the degradation of intracellular lipid droplets (LDs) *via* autophagy, also known as lipophagy (Fig. 4), were observed to promote lipid peroxidation and ferroptosis cell death in tumor cells (Bai et al., 2019b; Miess et al., 2018).

The fatty acid composition of PLs can influence sensitivity to ferroptosis in cancer. Melanoma cells exposed to the lymphatic environment, which is rich in oleic acid content, are able to escape from ferroptotic cell death and subsequently promote cancer metastasis spread. The suggested mechanisms by which oleic acid can act as a ferroptosis inhibitor relies on the reduction of the amount and/or density of membrane PUFAs that are available for oxidation (Ubellacker et al., 2020).

Nevertheless, the exact mechanism by which lipid peroxidation leads to ferroptotic cell death still needs to be elucidated. To date, data suggest cell death occurrence as the result of multiple events, including both direct damage to membrane PLs and the activation of downstream pathways (Lei et al., 2019; Stoyanovsky et al., 2019).

### C. Antioxidant systems

Among the several mediators of ferroptotic process, transcription factor nuclear factor erythroid 2 (NF-E2)-related factor 2 (NRF2) serves as a major regulator as many of the genes encoding for iron homeostasis systems (FTH1/FTL and FPN), lipid peroxide detoxification (GPX4), and GSH metabolism (glutamate-cysteine ligase catalytic subunit [GCLC]/GCLM and SLC7A11/xCT) are known to be NRF2 targets (Anandhan et al., 2020). Nevertheless, some NRF2 target genes, such as HO-1, play a dual role in ferroptosis (Adedoyin et al., 2018; Consoli et al., 2022; Kwon et al., 2015).

**FIG. 5.**
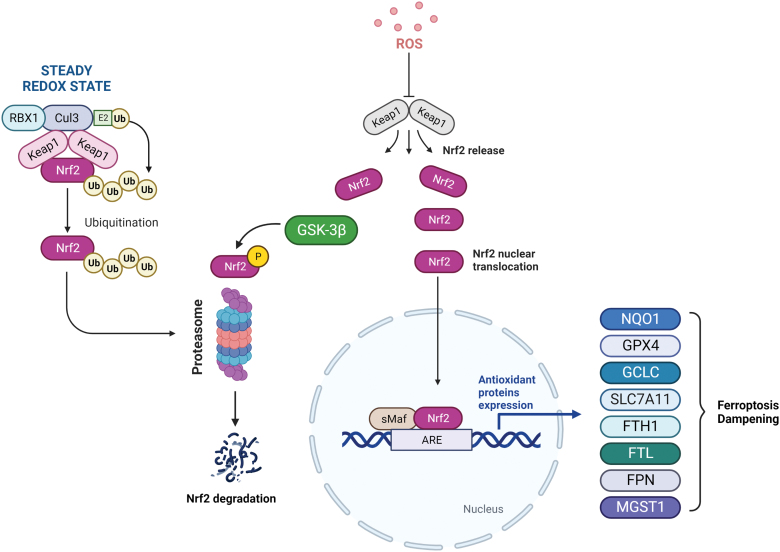
**NRF2 activation under oxidative stress conditions leads to its nuclear translocation, where it anchors ARE and heterodimerizes with sMaf proteins for the transcription of several antioxidant genes, resulting in ferroptosis dampening.** ARE, antioxidant response element; CUL3, cullin3; FTH1, ferritin heavy chain 1; FTL, ferritin light chain; GSK3β, Glycogen Synthase Kinase 3 Beta; Keap1, kelch-like ECH-associated protein 1; MGST1, microsomal glutathione S-transferase 1; NQO1, NAD(P)H Quinone Dehydrogenase 1; NRF2, nuclear factor erythroid 2 (NF-E2)-related factor 2; RBX1, ring box protein 1; ROS, reactive oxygen species; SLC7A11, solute carrier family 7 member 11; sMaf, small musculoaponeurotic fibrosarcoma proteins. Color images are available online.

Under basal conditions, NRF2 is the target of the kelch-like ECH-associated protein 1-cullin3-ring box protein 1 (Keap1-CUL3-RBX1) E3 ubiquitin ligase complex, which promotes its proteasomal degradation. Meanwhile, under oxidative stress conditions or in the case of mutations in Keap1, CUL3, or NRF2 itself, NRF2 ubiquitination cannot be executed and it is free to translocate into the nucleus to activate the transcription of antioxidant response element (ARE)-containing genes, many of which can affect and modulate the ferroptotic process (Fig. 5).

Further, NRF2 has also been proved to play a key role during tumorigenesis and also in cancer progression, including the resistance mechanism put into action by cells escaping programmed cell death (Rojo de la Vega et al., 2018). Thus, targeting NRF2 or its downstream effectors may represent a valid strategy to modulate ferroptosis in cancer cells.

Under physiological conditions, the antioxidant enzyme GPX4 is responsible for lipid peroxidation detoxification using the tripeptide GSH to reduce lipid peroxides to their alcohol form (LOH). GPX4 is the only isoform belonging to the GPX family for which membranes PL hydroperoxides and protein-thiol groups can act respectively as oxidizing and reducing substrates in the absence of GSH (Seiler et al., 2008; Yang et al. 2014b).

**FIG. 6.**
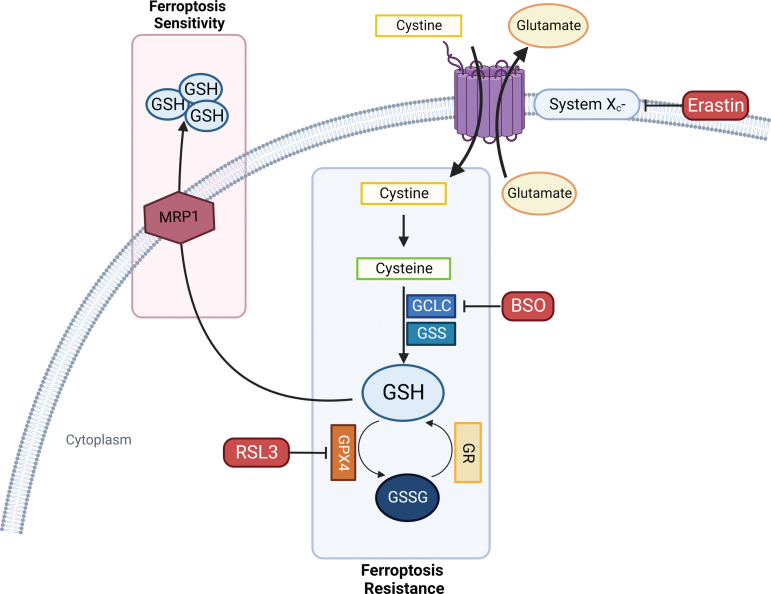
**GSH homeostasis mediates ferroptosis susceptibility.** BSO, buthionine sulfoximine; RSL3, RAS-selective lethal molecule 3. Color images are available online.

The Cys-GSH-GPX4-LOOH axis represents the foundation of the ferroptosis mechanism. *De novo* GSH synthesis must be provided by assembling it from cysteine (Cys), which can be obtained either from methionine through the trans-sulfuration pathway or from extracellular cystine import in the cytoplasm operated by the antiporter system xc^−^. Cystine is then reduced to cysteine to participate in GSH synthesis. Thus, system xc^−^ function serves as a major regulator of ferroptosis (Fig. 6) (Dixon et al., 2014; Hadian and Stockwell, 2020; Stockwell et al., 2017).

Metadata analysis showed that between the two main mechanisms of ferroptosis, direct GPX4 inhibitors were more cell-line selective than compounds that induce GSH depletion, which ultimately leads to loss of GPX activity. The study also identified reduced nicotinamide adenine dinucleotide phosphate (NADPH) as a biomarker for ferroptosis sensitivity (Kraft et al., 2020; Shimada et al., 2016).

#### 1. Glutamate-cysteine ligase

As GSH represents one of the most important regulators of ferroptosis, all the mechanisms involved in its synthesis and metabolism seem to highly affect the entire process (Fig. 6). GSH synthesis consists of two steps: The initial and limiting reaction leads to the formation of the glutamate-cysteine bond in the presence of GCL (glutamate-cysteine ligase) consuming ATP. Then, GSH synthetase (GSS) provides for Glu-Cys link with glycine to obtain the tripeptide GSH. Buthionine sulfoximine (BSO) acts as a GCL inhibitor, which, in turn, is able to indirectly inhibit GPX4 enzymatic activity triggering ferroptosis cell death (Shi et al., 2021).

#### 2. Multidrug resistance protein 1

Novel negative regulators of intracellular GSH content associated with ferroptosis sensitivity have been identified; among them, multidrug resistance protein 1 (MRP1) provides GSH cellular efflux and tends to collaterally sensitize cancer cells to ferroptosis (Cole, 2014) and its disruption has been linked to a strong inhibition of the ferroptotic process. Interestingly, MRP1 expression can be regulated by NRF2; indeed, NRF2 stabilization leads both to GSH increased cellular content and concomitantly to MRP1-dependent GSH efflux. However, GSH efflux has a higher impact in cellular homeostasis, driving cells to ferroptotic death (Fig. 6) (Cao et al., 2019).

Cancerous cells expressing high levels of MRP1 result in being resistant to most of the conventional chemotherapeutic drugs as it functions as an efflux pump; however, these findings suggest a role of MRP1-positive modulation that can be exploited to overcome drug resistance by sensitizing cells to a non-apoptotic cell death, placing the spotlight on ferroptosis.

#### 3. Reduced nicotinamide adenine dinucleotide phosphate

The aim of identifying biomarkers that are able to predict sensitivity to targeted agents as biomarkers implicated directly or indirectly to cancer development has widely been pursued in precision medicine. In this perspective, Stockwell and colleagues results first revealed that high NADP(H) levels are inversely correlated with sensitivity to ferroptosis inducers in cancer (Shimada et al., 2016). Thus, NADPH as a ferroptosis marker has started to gain attention and more in-depth studies have been conducted to better understand its role in this mechanism.

**FIG. 7.**
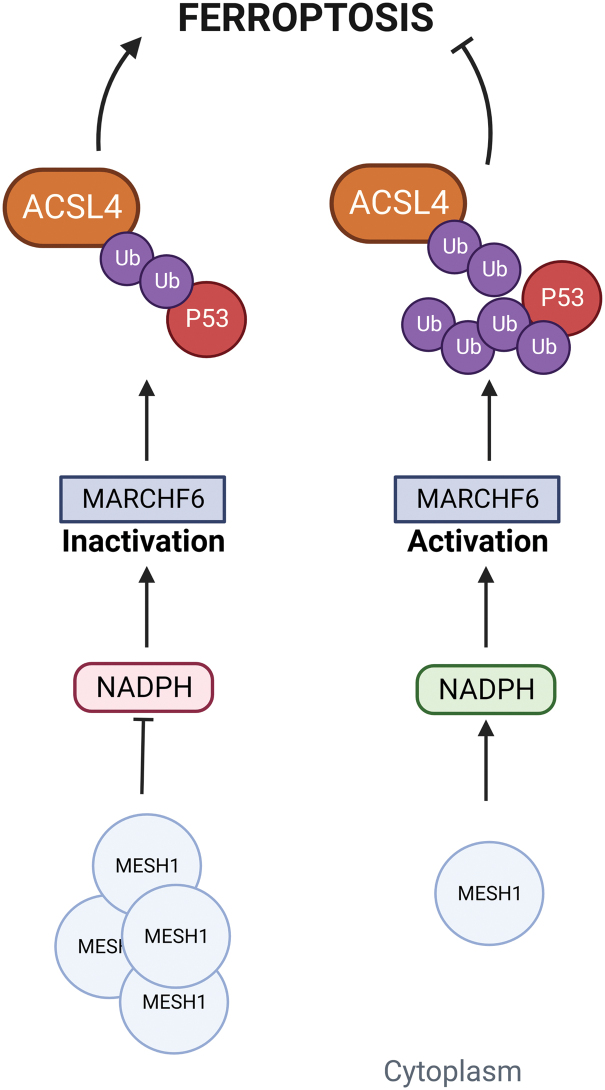
**NADPH as potential ferroptosis marker.** MARCHF6, Membrane Associated Ring-CH-Type Finger 6; MESH1, human Metazoan SpoT Homolog 1; Ub, ubiquitin. Color images are available online.

Ding et al. discovered human MESH1 (human Metazoan SpoT Homolog 1) implication in ferroptosis, describing it as a cytosolic NADPH phosphatase induced by erastin treatment. They were able to elucidate its molecular recognition of NADPH, suggesting a direct role of MESH1 in the execution of ferroptosis *via* NADPH degradation. Vice versa, MESH1 depletion and its NADPH phosphatase activity inhibition in ferroptosis-associated conditions enhance cell survival through NADPH preservation, GSH levels increase, and lipid peroxidation attenuation (Fig. 7) (Ding et al., 2020; Lin et al., 2021 ).

However, establishing NADPH role as a ferroptosis biomarker is still a small piece of the major puzzle represented by ferroptosis multiple-pathway-related regulation; indeed, specific regulators that can act as cellular NADPH sensors are largely unknown. Recently, data reported by Nguyen et al. identified Membrane Associated Ring-CH-Type Finger 6 (MARCHF6) E3 ubiquitin ligase as an NADPH sensor associated with ferroptosis regulation. The direct interaction between MARCHF6 activation region (MarA) located adjacent to the C-terminal MARCHF6 inhibitory region (MarI) and NADPH promoted E3 ligase activity of MARCHF6, resulting in ferroptosis dampening *via* the MARCHF6-dependent degradation of ferroptosis effectors ACSL4 and p53 (Mao and Gan, 2022; Nguyen et al., 2022).

#### 4. Ferroptosis suppressor protein 1

**FIG. 8.**
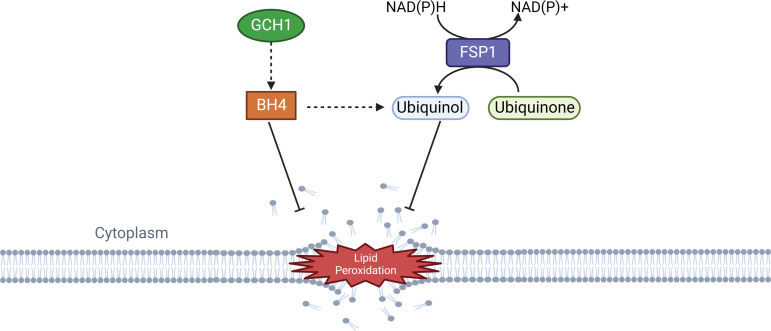
**GCH1-BH4 axis and FSP1 activity prevent lipid peroxidation and display anti-ferroptotic effects.** Color images are available online.

Recent findings identified ferroptosis suppressor protein 1 (FSP1), formerly known as apoptosis-inducing factor mitochondria associated 2 (AIFM2), as an effective ferroptosis-resistance factor (Fig. 8) (Bersuker et al., 2019; Doll et al., 2019). Indeed, FSP1 is recruited to the plasma membrane following myristoylation where it exerts its oxidoreductase activity catalyzing CoQ10 (also known as ubiquinone-10) reaction to ubiquinol by NADPH, a lipophilic radical scavenger that reduces lipid peroxides (Hadian, 2020; p. 1; Li and Li, 2020).

FSP1 can be activated by Peroxisome proliferator-activated receptor alpha (PPARα), which is under the control of the MDM2/MDMX complex and is able to regulate lipid cellular profile, independently from p53 (Venkatesh et al., 2020a). Interestingly, FSP1 negative regulation of ferroptotic process is independent of intracellular GSH and oxidizable fatty acid content, GPX4 enzymatic activity, and ACSL4 expression, revealing a non-canonical mechanism behind its function; thus, FSP1 inhibition could represent a valid strategy to sensitize cancer cells to ferroptosis (Shi et al., 2021).

Besides its oxidoreductase function, FSP1 can also initiate endosomal sorting complex required for transport-III (ESCRT-III)-dependent membrane repair to limit ferroptosis (Liu et al., 2022b). Recent studies have shown that the activation of ESCRT-III machinery leads to membrane repair by removing damaged parts of the cell membrane. ESCRT-III belongs to the family of ESCRT complexes, which is composed of five subcomplexes and plays a context-dependent role in membrane remodeling (Chen et al., 2021a; Motooka and Toyokuni, 2023). ESCRT-III confers resistance to ferroptotic cell death, allowing cell survival under stress conditions whereas knockdown of components of ESCRT-III machinery enhances ferroptosis (Dai et al., 2020).

#### 5. GCH1-BH4 axis

GTP cyclohydrolase-1 (GCH1) is a rate-limiting enzyme responsible for the production of tetrahydrobiopterin (BH4), which has antioxidant properties due to its function of generating reduced CoQ10 (ubiquinol), and rearranging lipids to reduce lipid peroxidation (Fig. 8). GCH1 expression level in cancer cell lines stratified susceptibility to ferroptosis, demonstrating a peculiar mechanism of ferroptosis protection that is not dependent from the GPX4/GSH system (Kraft et al., 2020). GCH1 overexpression proved to be effective in protecting cells against ferroptosis but not apoptosis and is only marginally effective against necroptosis; these observations indicate GCH1 selectivity in rescuing cells only from ferroptotic cell death (Wei et al., 2020).

#### 6. Microsomal glutathione S-transferase 1

MGST1 has recently been associated with ferroptosis cell death. MGST1 is a membrane-bound transferase, mainly located in the mitochondria, endoplasmic reticulum (ER) plasma membrane, and peroxisome, that takes part in cell defense processes against oxidative stress or electrophilic chemicals in an NRF2-dependent manner (Morgenstern et al., 2011).

Although it is known that MGST1 overexpression can inhibit oxidative stress and apoptotic cell death (Johansson et al., 2010; Zeng et al., 2020), its impact on ferroptosis has still to be elucidated. Recent studies have highlighted its role as a limiting factor during ferroptosis onset in pancreatic cancer cells (Dodson et al., 2021; Kuang et al., 2021). As MGST1 inhibition is a valuable way to overcome ferroptosis resistance *in vitro* and *in vivo*, this approach can represent an experimental basis for MGST1-mediated ferroptosis resistance, exploiting it as a potential target for cancer treatment.

Novel studies on crucial metabolic processes as nucleotide synthesis have shed light on another important link between GSH metabolism and ferroptosis. Indeed, Tarangelo et al. have reported that inhibition of nucleotide metabolism through p53 pathway can suppress ferroptotic cell death. It was observed that stabilization of wild-type p53 and induction of the p53 target gene cyclin dependent kinase inhibitor 1A (CDKN1A or p21) leads to decreased expression of the ribonucleotide reductase (RNR) subunits ribonucleotide reductase subunit 1 (RRM1) and RRM2, which exert their function reducing ribonucleotides to deoxyribonucleotides in a GSH-dependent manner (Sengupta et al., 2019; Tarangelo et al., 2022).

Thus, their results indicate that nucleotide synthesis regulation by the p53–p21 axis can provide another crucial link between GSH metabolism and ferroptosis susceptibility, even if an overactivation of cancer cells metabolism seems necessary to increase ferroptosis sensitivity.

### D. Tumor suppressor p53

In 2015, Jiang et al. first revealed a link between p53 and ferroptosis, highlighting a potential mechanism of cell sensitization to ferroptosis through p53 activity. Notably, it has been reported that a tight association exists between p53 and key metabolic pathways involved in ferroptosis (Liu et al., 2020). To date, many studies have been published that confirm p53 as a key regulator of both canonical and non-canonical ferroptosis pathways (Liu and Gu, 2022). p53 has been shown to display two opposite effects on ferroptosis, indeed it can promote or suppress ferroptosis depending on cellular conditions (Kang et al., 2019).

Under normal conditions, p53 can increase tumor cell sensitivity to ferroptosis, promoting cell death. However, on exposure to stresses such as cysteine deprivation, p53 hinders ferroptosis (Friedmann Angeli et al., 2019; Zhang et al., 2022b). To further elucidate the mechanism of p53 regulation, Wang et al. (2016) conducted a screening to uncover previously unknown modifications of p53. The study elucidated the fundamental role of p53 acetylation, which affects p53 ability to transcriptionally regulate its metabolic targets, such as TP53-induced glycolysis and apoptosis regulator (TIGAR), Glutaminase 2 (GLS2), and SLC7A11, and induce ferroptosis and tumor suppression. SLC7A11 has been identified as a direct p53 target gene (Liu et al., 2022c).

Mechanistically, p53 promotes the activity of ALOX12 through SLC7A11 inhibition. SLC7A11 directly binds ALOX12, preventing its interaction with the substrate, PUFAs, including those esterified in membranes. SLC7A11 downregulation by p53 leads to ALOX12 release and activation and consequent initiation of ferroptosis (Chu et al., 2019; Liu and Gu, 2022). Beyond downregulating SLC7A11, p53 promotes ferroptosis through the regulation of other metabolic pathways.

For instance, Ou et al. (2016) demonstrated that p53 transactivates spermidine/spermine N1-acetyltransferase 1 (SAT1), which is a rate-limiting enzyme in polyamine catabolism, resulting in a reduction of xenograft tumor growth. Interestingly, SAT1 induction was associated with increased lipid peroxidation and ferroptosis activation, mainly through SAT1-dependent ALOX15 upregulation. Thus, p53/SAT1/ALOX15 axis partially contributes to p53-mediated ferroptosis and tumor suppression. In addition, p53 can facilitate glutaminolysis, which, in turn, is able to promote ferroptosis. Indeed, p53-mediated activation of GLS2, a mitochondrial enzyme catalyzing the first step of glutamine catabolism, can boost ferroptosis.

On the contrary, a number of studies reported that prolonged stabilization of wild-type p53 renders many cancer cells less sensitive to system xc^−^ inhibition or direct cystine deprivation-induced ferroptosis (Tarangelo et al., 2018; Xie et al., 2017). Reduction of sensitivity to ferroptosis was associated with p21 activation and intracellular GSH levels preservation (Venkatesh et al., 2020b). However, it is still unclear how activation of the p53-p21 axis may affect cellular cystine import and *de novo* GSH synthesis.

Dipeptidyl-peptidase-4 (DPP4), a multiple functional protease that plays an important role in mediating cell death, seems to be involved in p53-mediated anti-ferroptotic effect. p53 bond to DPP4 regulates the subcellular localization of DPP4 but not its protein levels. In the absence of p53, DPP4 forms a complex with NADPH oxidase 1 (NOX1), which contributes to plasma membrane lipid peroxidation and ferroptosis. After binding p53, DPP4 is sequestered in a nuclear enzymatic inactive pool, which leads to NOX1 dissociation and decreased lipid peroxidation and ferroptosis. Interestingly, depletion or inhibition of p53 only enhances ferroptosis induced by system xc^−^ inhibitors (*e.g*., erastin) but not ferroptosis induced by GPX4 inhibitors (*e.g*., RSL3 or FIN56) (Xie et al., 2017).

Moreover, it was observed that CRISPR/Cas9 technology-mediated p53 depletion enhanced cell sensitivity to ferroptosis, supporting a pro-survival function of p53 in ferroptosis (Kang et al., 2019).

### E. Non-coding RNAs

Non-coding RNAs (ncRNAs) are classified as a group of RNAs exempt from translation into polypeptides that are able to tune the expression of genes involved in various physio-pathological conditions (Valashedi et al., 2022).

MicroRNAs (miRNAs) are a class of ncRNAs counting about 22 nucleotides, whereas long noncoding RNAs (lncRNAs) definition include more than 200 nucleotides transcripts that are not translated into proteins. For a long time, they have been considered as “junk RNA,” since their actual role in cellular processes was not clear. In recent years, the interest toward lncRNAs has increased as new findings point out to their involvement in cancer occurrence and development, indeed they seem to affect cell proliferation (Jie et al., 2020; Wang et al., 2020b) and differentiation (Gao et al., 2019a; Ponzio et al., 2017). Empirical evidence has shown ncRNAs participation in cancer development, exploiting the modulation of different forms of programmed cell death, including apoptosis, autophagy, necroptosis, and pyroptosis (Jiang et al., 2021a; Shirjang et al., 2019).

Intriguingly, it was observed that lncRNAs can regulate lipid metabolism in cancerous cells and therefore be able to modulate ferroptosis (D'Souza et al., 2020; Farooqi et al., 2023; Huarte, 2015). Studies have reported ferroptosis promotion by the cytosolic lncRNA P53RRA through the nuclear sequestration of p53, leading to iron and lipid ROS accumulation in lung adenocarcinoma (Mao et al., 2018; Mou et al., 2019). Other lncRNAs have been observed to be involved in ferroptosis onset in cancer acting as downregulators of NRF2, such as lncRNA KRAL (Wu et al., 2018), lncRNA GABPB1-AS1 (Qi et al., 2019), and lncRNA MALAT1 (Amodio et al., 2018).

In addition, several miRNAs act as modulators of the ferroptotic process, both promoting and inhibiting it, such as miR-6852 (Wang et al., 2019a), miR-7-5p (Tomita et al., 2019), miR-76 (Zhang et al., 2020a), miR-9 (Zhang et al., 2018), miR-137 (Luo et al., 2018), and miR-17-92 (Xiao et al., 2019). Several miRNAs also regulate post-transcriptionally SLC7A11 levels, which is post-translationally stabilized by an interaction with CD44v9 (a variant of the CD44 stemness marker of several cancers) leading to inhibition of proteosomal degradation in cancer (Jyotsana et al., 2022).

## IV. Ferroptosis in Cancer

Ferroptosis has been associated with several physio-pathological processes, including cancer; thus, ferroptosis induction holds great potential as a novel therapeutic strategy for cancer treatment, especially for those types no longer responding to conventional chemotherapy. The peculiar metabolism of cancerous cells, associated with high levels of ROS, and specific mutations drive susceptibility to ferroptosis in some tumors, highlighting some soft spots that can represent valuable targets for cancer treatment (Lei et al., 2022; Viswanathan et al., 2017).

The ferroptosis characterizing mechanisms in solid tumors seem to be shared with hematological cancers such as leukemia, lymphoma, and multiple myeloma (Chen et al., 2022c; Schmitt et al., 2021; Zhao et al., 2021).

**FIG. 9.**
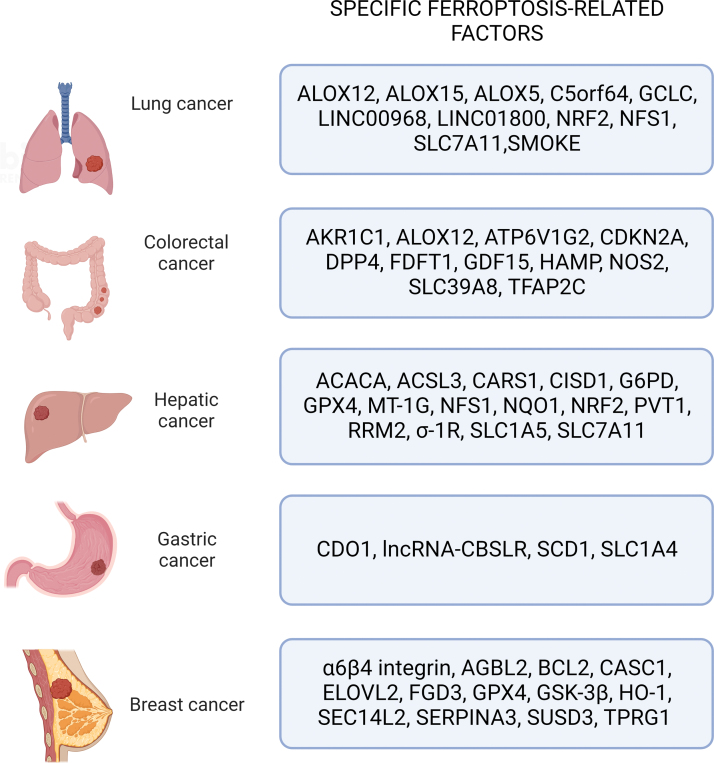
**Representation of the principal ferroptosis-related factors specific for each type of cancer reported.** σ_1_R, sigma-1 receptor; ACACA, acetyl-coa carboxylase alpha; AGBL2, AGBL Carboxypeptidase 2; AKR1C1, aldo-keto reductase family 1 member C1; ATP6V1G2, ATPase H+ transporting V1 subunit G2; BCL2, B-cell lymphoma 2; C5orf64, lncRNA Chromosome 5 Putative Open Reading Frame 64; CARS1, Cysteinyl-TRNA Synthetase 1; CASC1, cancer susceptibility candidate 1; CDKN2A, cyclin dependent kinase inhibitor 2A; CDO1, cysteine dioxygenase 1; ELOVL2, elongation of very-long-chain fatty acids-like 2; FDFT1, farnesyl-diphosphate farnesyltransferase 1; FGD3, facio-genital dysplasia 3; G6PD, Glucose-6-Phosphate Dehydrogenase; GDF15, growth differentiation factor 15; HAMP, hepcidin antimicrobial peptide; LINC00968, Long Intergenic Non-Protein Coding RNA 968; LINC01800, Long Intergenic Non-Protein Coding RNA 1800; lncRNA, long noncoding RNA; MT-1G, metallothionein-1G; NFS1, cysteine desulfurase; NOS2, nitric oxide synthase 2; PVT1, lncRNA plasmacytoma variant translocation 1; RRM1, ribonucleotide reductase subunit 1; SEC14L2, SEC14 Like Lipid Binding 2; SERPINA3, Serpin Family A Member 3; SLC1A4, Solute Carrier Family 1 Member 4; SLC1A5, Solute Carrier Family 1 Member 5; SLC39A8, solute carrier family 39 member 8; SUSD3, Sushi Domain Containing 3; TFAP2C, transcription factor AP-2 gamma; TPRG1, Tumor Protein P63 Regulated 1. Color images are available online.

Ferroptosis was observed to induce cell death in several cancers. Here, we will discuss ferroptosis-related factors implicated in the five deadliest types of cancers worldwide according to most recent estimations (Fig. 9) (Siegel et al., 2022).

### A. Lung cancer

Lung cancer is one of the most common causes of cancer-related death in the world. The two main types of lung cancer are non-small-cell lung cancer (NSCLC) and small-cell lung cancer (SCLC), which account for ∼85% and 15% of all newly diagnosed lung cancers, respectively (Oser et al., 2015; Wu et al., 2021a). Bioinformatic prediction studies by Liu et al. (2021c) have reported the involvement of ferroptosis-related genes, such as ALOX5, GCLC, and SLC7A11, in NSCLC progression and prognosis.

Several cellular key regulators, such as p53, NRF2, NFS1 (cysteine desulfurase), LSH (lymphoid-specific helicase), and ncRNAs, act as modulators of ferroptosis sensitivity in lung cancer. In particular, ferroptosis can be enhanced by ALOX15 and ALOX12 activation as downstream effectors of the p53 pathway, which, in turn, can cause SLC7A11 inhibition in H1299 lung cancer cells (Chu et al., 2019; Ou et al., 2016).

Further, NRF2 and KEAP1 mutations have been observed in a significant percentage of patients with NSCLC, leading to adaptive response and drug resistance, also determining a certain resistance to erastin-induced cell death (Gai et al., 2020; Hayes and McMahon, 2009).

The ubiquitous iron-sulfur (Fe-S) clusters are adaptable cofactors required for several survival processes. The initial step of the Fe-S cluster biosynthesis is mediated by mitochondrial NFS1, which releases sulfur from cysteine. NFS1 is found overexpressed in patients with lung adenocarcinoma, and increased levels of NFS1 promote growth of primary lung tumor cells *in vitro*, whereas NFS1 knockdown improves anticancer activity of ferroptosis-inducing compounds in lung cancer cells (Alvarez et al., 2017).

Among the factors associated with poor prognosis in lung cancer patients, it is worth mentioning LSH activity and some lncRNAs contribution as they have been associated with ferroptosis. LSH can aggravate cancer due to its ability of inhibit ferroptosis *via* the activation of metabolism-related genes as solute carrier family 2 member 1 (SLC2A1/GLUT1) and FADS2 both *in vitro* and *in vivo* (Jiang et al., 2017; Yang et al., 2019). Indeed, FADS2 mediates the production of MUFAs, which can reduce PUFAs-induced ferroptosis in lung cancer (Magtanong et al., 2019).

Several ferroptosis-related lncRNAs, such as lncRNA Chromosome 5 Putative Open Reading Frame 64 (C5orf64), Long Intergenic Non-Protein Coding RNA 1800 (LINC01800), and Long Intergenic Non-Protein Coding RNA 968 (LINC00968), have been observed to be protective factors in lung cancer patients (Lu et al., 2021). In addition, LINC00472/P53RRA functions as a tumor suppressor complex and it is downregulated in multiple cancers, including lung cancer. Cytosolic LINC00472 undermines p53 interaction with G3BP stress granule assembly factor 1 (G3BP1), resulting in increased levels of p53 in the nucleus and ferroptosis promotion (Mao et al., 2018).

Moreover, whole cigarette smoke condensates were observed to induce ferroptosis in human bronchial epithelial cells, enabling cigarette smoking-induced chronic obstructive pulmonary disease pathogenesis and increasing the risk of lung cancer insurgence (Park et al., 2019). Further, lung epithelial cells exposed to cigarette smoke release damage-associated molecular patterns (DAMPs) and pro-inflammatory cytokines that have been associated with ferritinophagy-dependent iron accumulation and ferroptosis (Yoshida et al., 2019).

### B. Colorectal cancer

Ferroptosis relevance in colorectal cancer (CRC) has been explored in the past decade.

A key factor associated with the ferroptosis regulation in CRC was the DPP4, which is under the control of p53. Xie et al. (2017) reported that CRC cells were resistant to ferroptosis due to the inhibition of DDP4 activity mediated by p53 in a transcription-independent way.

Recent studies have unraveled a link between copper (Cu^2+^) overload and inhibition of CRC proliferation both *in vitro* and *in vivo*. Gao et al. (2021) observed mitochondrial Cu^2+^ overload after treatment with the chelator elesclomol associated with a decrease in ATP7A (copper-transporting ATPase 1) expression and degradation of SLC7A11, resulting in Cu^2+^ retention and extremely increased oxidative stress, which leads to ferroptosis in CRC cells. Interestingly, Cu^2+^ trafficking seems to be involved in ferroptosis regulation in CRC.

Indeed, cancer cells upregulate several Cu^2+^ chaperones such as the copper chaperone for SOD1 (CCS), which binds cytosolic Cu^2+^ and transfers it to SOD1, and pharmacological disruption of this process can have therapeutic effects on cancer as it reduces uncontrolled cell proliferation (Li et al., 2019b; Rae et al., 1999; Wang et al., 2015). Although the contribution of Cu^2+^ is not specific to CRC, recently it has been reported that Cu-nanoparticles used for photodynamic therapy presented a safe and promising clinical application prospect for deep-seated tumors and effectively inhibited CRC cell proliferation by inducing ferroptosis (Zhou et al., 2023).

These findings seem to point out to a solid link between Cu^2+^ homeostasis and ferroptosis in CRC, even though more in-depth studies are needed to better understand the mechanism by which it can actually affect and/or modulate ferroptotic cell death. A predictive model on the relationship between ferroptosis and prognosis of CRC patients was established by Shao et al. screening differential ferroptosis-related genes from The Cancer Genome Atlas (TCGA) dataset.

The study was able to determine ten ferroptosis-related genes signature in CRC: transcription factor AP-2 gamma (*TFAP2C*), solute carrier family 39 member 8 (*SLC39A8*), nitric oxide synthase 2 (*NOS2*), hepcidin antimicrobial peptide (*HAMP*), growth differentiation factor 15 (*GDF15*), farnesyl-diphosphate farnesyltransferase 1 (*FDFT1*), cyclin dependent kinase inhibitor 2A (*CDKN2A*), *ALOX12*, aldo-keto reductase family 1 member C1 (*AKR1C1*), and ATPase H+ transporting V1 subunit G2 (*ATP6V1G2*). These genes were then classified depending on their molecular activity in transcription factors (*TFAP2C*), energy (*GDF15*, *FDFT1*, *AKR1C1*, *ATP6V1G2*), iron (*SLC39A8*, *HAMP*, *CDKN2A*), and oxidative metabolism modulators (*NOS2*, *ALOX12*) (Shao et al., 2021a).

### C. Hepatic cancer

Hepatic cancer is one of the most frequent causes of cancer death worldwide and patients often get the diagnosis once in advanced stages, contributing to its poor prognosis. Among the liver cancer cases reported, more than 90% are HCCs (Anwanwan et al., 2020).

Sorafenib is a multikinase inhibitor and it has been used as gold standard for the treatment of advanced HCC. In 2013, researchers proved that sorafenib is able to induce ferroptosis and since then it has been classified as a ferroptosis inducer (Louandre et al., 2013). However, recent evidence reported by Zheng et al. proved that sorafenib actually fails in triggering ferroptosis in several cancers, including four different hepatoma cell lines. Thus, the usage of sorafenib in future ferroptosis-related studies should take that into account.

More and more studies have highlighted the pivotal role of ferroptosis in HCC and the strict correlation between some ferroptosis modulators activity in cancer development such as p53, retinoblastoma (Rb) protein, and NRF2 (Jennis et al., 2016; Louandre et al., 2015; Nie et al., 2018; Sun et al., 2016b; p. 2).

The increasing acquired resistance to sorafenib in HCC patients has pushed research to look for the mechanism behind this process to reverse it or avoid it, also exploiting novel knowledge about ferroptosis cell death. Recent evidence suggested an important role of metallothionein-1G (MT-1G) as a negative regulator of ferroptosis whose genetic and pharmacological inhibition enhance sorafenib anticancer activity both *in vitro* and in tumor xenograft models leading to GSH depletion and lipid peroxidation without altering iron cellular content (Sun et al., 2016a).

Interestingly, the sigma-1 receptor (σ_1_R) seems to be involved in ferroptosis mechanism as haloperidol (σ_1_R antagonist) was observed to promote erastin-induced ferroptotic cell death in HCC cells (Bai et al., 2019a; Bai et al., 2017). Transcription factors hypermethylated in cancer 1 (HIC1) and hepatocyte nuclear factor 4 alpha (HNF4A) were identified by Zhang et al. (2019a) as crucial modulators of ferroptosis-related factors, with stimulating and suppressing activity, respectively, and correlated with tumor stage in liver cancer: Worst prognostic outcomes were associated with low HIC1 and high HNF4A levels.

HIC1 acts as a tumor suppressor inhibiting cell growth, migration, and survival; on the other hand, HNF4A is critical for liver development and is upregulated in liver cancer (Dill et al., 2013; Parviz et al., 2003; Xu et al., 2001). However, the exact role of HNF4A is still not clear since on the contrary its downregulation was seen to be associated with poor prognosis in renal clear cell carcinoma (Gao et al., 2019b). Zang et al. study suggests that ferroptosis stimulation leads to an altered balance between HIC1 and HNF4A that can compromise cancer development and may be useful as a potential new strategy for liver cancer treatment.

As previously reported for CRC, a study by Liang et al. (2020) established a prognostic model composed by 10 ferroptosis-related genes also for HCC, including Acetyl-CoA Carboxylase Alpha (*ACACA*), Acyl-CoA Synthetase Long Chain Family Member 3 (*ACSL3*), *CISD1*, Cysteinyl-TRNA Synthetase 1 (*CARS1*), Glucose-6-Phosphate Dehydrogenase (*G6PD*), *GPX4*, NAD(P)H Quinone Dehydrogenase 1 (*NQO1*), *NFS1*, *SLC7A11*, and Solute Carrier Family 1 Member 5 (*SLC1A5*).

Recently, RRM2 (Ribonucleotide Reductase Regulatory TP53 Inducible Subunit M2B) was discovered as a ferroptosis-related tumor biomarker in liver cancer; indeed, Tang et al. (2021a) demonstrated its anti-ferroptotic activity *via* a sustained GSH production. Moreover, the correlation between high RRM2 serum levels and higher tumor stage in liver cancer makes it a potential predictive marker of ferroptosis susceptibility and a targetable factor for cancer treatment (Yang et al., 2020; p. 2).

Work from He et al. (2021) unraveled ketamine ability to induce ferroptosis in hepatic cancer *via* regulation of the lncPVT1/miR-214-3p/GPX4 axis. LncRNA plasmacytoma variant translocation 1 (PVT1) is overexpressed and implicated in several types of cancer (Chen et al., 2019; p. 1; Liu and Xu, 2020; Ren et al., 2019; Zhou et al., 2020), and specifically it was demonstrated that its expression can regulate GPX4 expression levels through miR-214-3p suppression to favor ferroptosis inhibition in liver cancer.

Indeed, ketamine treatment negatively affected lncPVT1 levels, leading to ferroptosis onset in liver cancer cells and proving the potential benefits of targeting lncPVT1/miR-214-3p/GPX4 axis as novel therapeutic approach.

### D. Gastric cancer

Adenocarcinomas represent the majority of gastric tumors across the globe, unfortunately the prognosis is discouraging with an average of 5-year survival rate in <20% of patients due mainly to no clinical evidence in the early stages, which leads to late diagnosis (Correa, 2013). Pathological conditions such as anemia, autoimmune gastritis, and low FT levels (Fonseca-Nunes et al., 2015) associated with iron poor absorption have been indicated as risk factors for gastric cancer (GC) insurgence (Kamada et al., 2022; Nomura et al., 1992; Prá et al., 2009). Based on this evidence, several studies have been carried out to understand the correlation between GC and ferroptosis (Gu et al., 2022).

GC patients were found to display SCD1 overexpression, which was associated with SCD1-dependent increase of proliferation-related marker (PCNA), anti-apoptosis marker survivin, and anti-ferroptosis markers SLC7A11 and GPX4. Based on these observations, Wang et al. (2020a) demonstrated by using *in vitro*, *in vivo*, and *in silico* methods that SCD1 promote proliferation of GC cells and tumor growth concomitantly with protecting them from ferroptotic cell death.

According to the findings of Hao et al., human cysteine dioxygenase 1 (CDO1) plays a key role in modulating ferroptosis in GC. Indeed, CDO1 is a non-heme iron metalloenzyme that catalyzes the reaction of cysteine oxidation to its sulfinic acid, leading to the formation of taurine (Parham et al., 1991). Increased cysteine levels can exert cytotoxic activity due to the concomitant increase of sulfinic acid and sulfites (Poltorack and Dixon, 2022); however, overexpression of CDO1 may reduce cysteine availability, which results in GSH levels reduction, ROS increase, and, ultimately, ferroptosis induction and cell death.

Conversely, inhibition of CDO1 activity leads to ferroptosis resistance, proving its significant influence on this programmed cell death (Hao et al., 2017). Recently, large-scale clinical studies have identified crucial ferroptosis-related genes as potential biomarkers for GC to predict immune-antitumoral drug responses (Jiang et al., 2021b; Shao et al., 2021b). Interestingly, competing endogenous RNAs (ceRNAs) network analysis demonstrated a correlation between ZFP36, TGFBR1, MYB, SP1, and Solute Carrier Family 1 Member 4 (SLC1A4) ferroptosis-related genes and ceRNA processes affecting the tumor microenvironment (Liu et al., 2021b).

Moreover, lncRNA-CBSLR was found to be a ferroptosis modulator in GC cells *via* the YTH N6-methyladenosine RNA-binding protein 2 (YTHDF2)/Cystathionine beta-synthase (CBS)/ACSL4 axis (Yang et al., 2022b).

### E. Breast cancer

BC is the malignant tumor with the highest mortality in women. Unstoppable cancer progression is mainly derived from resistance to apoptosis, which can be developed following conventional apoptotic-inducing drug protocols. Therefore, many researchers have been focusing their attention on new drugs or models that can overcome drug resistance (Sui et al., 2022). Iron homeostasis is essential for cellular metabolism and it is particularly involved in tumor growth, especially in those types with high malignancy.

Iron is crucial for ROS production and can contribute either to cell proliferation or to cell death in BC, suggesting the existence of a delicate balance of pro and anti-oxidant conditions within the cell that determines its own fate (Dixon and Stockwell, 2014; Li et al., 2020d).

Several studies have elucidated the pivotal role of GPX4 in ferroptosis; indeed, GPX4 inhibition results in ferroptotic cell death in resistant BC as they completely rely on its antioxidant and detoxifying activity (Hangauer et al., 2017). According to Wu et al. (2020a), Glycogen Synthase Kinase 3 Beta (GSK3β) overexpression in BC cells and *in vivo* BC xenograft can boost erastin-induced ferroptosis. GSK3β is an essential element that mediated downregulation of the antioxidant cellular defense through NRF2 ubiquitination and subsequent degradation (Armagan et al., 2019). Indeed, GSK3β antagonizes NFR2 function and compromises NRF2-dependent antioxidant pathways, inducing ferroptosis and revealing a promising therapeutic approach for BC treatment.

Co-treatment of siramesine and lapatinib triggered ferroptosis in different breast carcinoma cell lines (MCF-7, MDA-MB-231, ZR-75, and SKBr3) *via* increased iron levels and ROS production as reported by Ma et al. (2016). Moreover, it was proved that ferroptosis induction by GPX4 inhibition can resensitize gefitinib-resistant triple negative breast cancer (TNBC) cells to gefitinib (Song et al., 2020; p. 4). Another study reported metformin capacity of inducing ferroptosis by decreasing SLC7A11 protein stability through UFMylation process inhibition.

In addition, SAS and metformin were observed to induce a synergistic effect reducing invasiveness of BC through the activation of the ferroptotic process (Yang et al., 2021).

An intriguing discovery was the correlation between ferroptosis and cell adhesion and density in breast tumor. The α6β4 integrin has been described as an active participant in cancer progression, and it contributes to cell adhesion mechanisms (Chen et al., 2009; Lipscomb and Mercurio, 2005). Brown et al. recently described extracellular matrix (ECM) detachment as a physiologic trigger of ferroptosis in BC cells due to consequent GPX4 inhibition; however, α6β4 integrin can help cells escape this process by activating STAT3 and suppressing ACSL4 expression, exerting a protective function for membrane lipids integrity (Brown et al., 2017).

It was observed that α6β4 integrin not only preserved membrane lipid integrity, preventing cells from undergoing ferroptosis following ECM detachment but also could shield adherent epithelial and carcinoma cells from erastin-induced ferroptosis. In the absence of α6β4, lipid peroxidation levels markedly increase, restoring cells susceptibility to ferroptosis (Brown et al., 2018).

Recent studies have highlighted the curcumin anti-tumor effect through ferroptosis induction in BC cells (Li et al., 2020b). Curcumin-induced ferroptosis was also observed to be related to HO-1 upregulation and activation, demonstrating together with the administration of its substrate hemin that it can modulate and drive ferroptosis in TNBC cells. Interestingly, MDA-MB 231 TNBC cells seem to be more sensitive to ferroptosis inducers as erastin and then less aggressive hormone-dependent MCF-7 cell line (Consoli et al., 2022).

Zhang et al. (2021b) were able to identify a novel ferroptosis-related lncRNA signature that could predict the prognosis of BC patients. The study contributed to unravelling the relationship between the expression of ferroptosis-related lncRNAs in BC and patient prognosis, establishing a correlation between 11 ferroptosis-related lncRNAs and oxidative stress of BC patients. Thus, ferroptosis-related lncRNAs may have a potential role as therapeutic targets for BC.

A year later, Yin and Tang (2022) found nine ferroptosis-related genes (B cell lymphoma 2 [*BCL2*], Sushi Domain Containing 3 [*SUSD3*], Serpin Family A Member 3 [*SERPINA3*], AGBL Carboxypeptidase 2 [*AGBL2*], SEC14 Like Lipid Binding 2 [*SEC14L2*], elongation of very-long-chain fatty acids-like 2 [*ELOVL2*], facio-genital dysplasia 3 [*FGD3*], cancer susceptibility candidate 1 [*CASC1*], Tumor Protein P63 Regulated 1 [*TPRG1*]) with prognostic value and contributed to the construction of genetic prognostic models, exploiting the relationship between ferroptosis calculated score and BC patients prognosis.

## V. Natural Compounds as Ferroptosis Inducers

### A. Alkaloids

Alkaloids are a vast class of compounds containing a nitrogen atom and endowed with numerous biological effects, including antiarrhythmic, analgesic, antimalarial, anesthetic, and anticancer activities (Mondal et al., 2019). Recently, it has been reported that compounds belonging to this chemical class possess pro-ferroptotic properties in different cancer cell lines.

#### 1. Capsaicin

##### i. *In vitro*

Capsaicin is a benzyl alkaloid derived from homovanillic acid whose presence is mainly detectable in plants belonging to the *Solanaceae* family, *Capsicum* genus (Chapa-Oliver and Mejía-Teniente, 2016). The therapeutic properties of capsaicin as a cardioprotective, gastroprotective, antihypercholesterolemic, analgesic, and antioxidant compound have been extensively studied (Luo et al., 2011; Srinivasan, 2016). In addition, it was found that capsaicin also possesses anticancer and antiangiogenic effects and lack of toxicity to healthy cells (Clark and Lee, 2016; Min et al., 2004).

Interestingly, Liu et al. (2022d) reported on the ability of capsaicin to induce ferroptosis in NSCLC through the SLC7A11/GPX4 pathway. Specifically, the cell viability of the A549 and NCI-H23 cell lines was reduced in a concentration and time-dependent manner through an increase of the total and ferrous iron content, decrease of the SLC7A11 and GPX4 messenger RNA (mRNA) levels, and decrease of the GSH content. Similar results were also detected in two different glioblastoma cell lines (U87-MG and U251) (Hacioglu and Kar, 2023). Indeed, capsaicin treatment determined ferroptosis in these cell lines, increasing ACSL4, 5-hydroxyeicosatetraenoic acid (5-HETE), total oxidant status (TOS), MDA levels, and lactate dehydrogenase (LDH) activity and decreasing GPX, total antioxidant status (TAS), and GSH levels.

#### 2. Lycorine

##### i. *In vitro*

Lycorine is a pyrrolophenanthridine alkaloid whose natural source is represented in plants of the *Amaryllidaceae* family. This molecule displayed antiviral, antibacterial, antiparasitic, anti-inflammatory, and antitumoral effects. Roy et al. (2018) reviewed the biological effects of this molecule as well as the chemical modifications responsible for tuning its antitumoral pharmacological effect. The role of lycorine in ferroptosis induction has not been well documented. Very recently, Du et al. (2021) addressed the role of lycorine in ferroptosis induction in renal cell carcinoma (Caki-1, A498, and 786-O cell lines).

Similar to capsaicin, lycorine treatment caused the decrease of GPX4 and reduced the GSH/GSSG (GSH disulfide) ratio, whereas the ACSL4, 5-HETE, 12-HETE, 15-HETE, and MDA levels were increased. In addition, co-administration of ferrostatin-1 (Fer-1) reversed the effects exerted by lycorine, suggesting that cell death occurred through ferroptosis induction.

#### 3. Piperlongumine

##### i. *In vitro*

Piperlongumine is an alkaloid found in *Piper longum* characterized by the presence of an amide function (Li et al., 2022a). The molecule has been described as a thioredoxine reductase 1 (TXNRD1) inhibitor through the formation of a covalent bond with a selenocysteine residue of the enzyme (Warner et al., 2000; Yang et al., 2022c). TXNRD1 is an enzyme with antioxidant properties and its overexpression is often associated with cancer progression and reduced sensitivity to high ROS levels (Gencheva and Arnér, 2022).

In HCT-116 colon cancer cells, piperlongumine failed in inducing ferroptosis; however, it sensitized cancer cells to erastin-dependent lipid peroxidation (Yang et al., 2022c). Conversely, piperlongumine strongly increased ROS and mRNA HO-1 levels in MIAPaCa-2 and PANC-1 pancreatic cancer cells, triggering ferroptosis. Interestingly, these effects were further enhanced when cells were co-treated with cotylenin A, a terpenoid natural compound, and/or SAS, whereas no significant cytotoxicity was observed in mouse embryonic fibroblasts treated with the three-drug combination (Yamaguchi et al., 2018).

#### 4. Sanguinarine

##### i. *In vitro*

*Sanguinaria canadensis* is the major source of sanguinarine, a quaternary benzophenanthridine alkaloid with anticancer effects (Galadari et al., 2017). Very recently, sanguinarine was shown to induce apoptosis and ferroptosis in human cervical cancer (HeLa) cells (Alakkal et al., 2022). Both regulated forms of cell death were evoked through sanguinarine-dependent ROS production, especially H_2_O_2_. Specifically, apoptosis was confirmed by poly(ADP-ribose) polymerase (PARP) cleavage and caspase activation. Z-VAD-fmk, a caspase inhibitor, partially prevented HeLa cancer cell death.

At the same time, cell treatment with sanguinarine caused increased lipid peroxidation and iron levels and decreased GSH and SLC7A11. Treatment with Fer-1, deferoxamine (DFO), or trolox prevented ferroptosis. Interestingly, cell treatment with an ROS inhibitor was effective in inhibiting both apoptosis and ferroptosis, whereas the selective inhibition of apoptosis prevented ferroptosis and vice versa. For this reason, the authors of this work speculated that the sanguinarine anticancer effect is due to the crosstalk between apoptosis and ferroptosis throughout the generation of H_2_O_2_.

#### 5. Solasonine

##### i. *In vitro*

Solasonine is an oxaspiro and azaspiro glycoalkaloid found in some plants of the *Solanaceae* family with antiproliferative effects in gastric (Li et al., 2022f; Zhang et al., 2020b), bladder (Dong et al., 2022), hepatic (Pham et al., 2019), glioma (Wang et al., 2017), and colon cancer (Lee et al., 2004). In A549 and Calu-1 lung adenocarcinoma cancer cell lines, solasonine caused cell death by ferroptosis with IC_50_ values of 15.08 and 21.59 μ*M*, respectively. The mechanism of action was explained by increased mitochondrial membrane depolarization and ROS production, the accumulation of lipids that underwent peroxidation, high Fe^2+^ levels, and decreased GPX4, SLC7A11, and intracellular cysteine levels (Zeng et al., 2022).

##### ii. *In vivo*

In HCC, solasonine treatment determined ferroptosis in a xenograft model disrupting the activity of the GSH redox system and therefore increasing ROS concentration (Jin et al., 2020). In addition, PANC-1 and CFPAC-1 pancreatic cancer cells were susceptible to apoptosis and ferroptosis when treated with solasonine concentrations ranging from 5 to 50 μ*M* (Liang et al., 2022). Of interest, the authors of this work were able to identify the intracellular target of solasonine and proposed a mechanism of action that finally evoked cell death and blockage of metastasis both *in vitro* and *in vivo*.

Specifically, the selected pancreatic cancer cells upregulated the mRNA expression of the transcription factor activating enhancer binding protein 2 alpha (TFAP2A), a protein involved in tumor progression and poor prognosis. Interestingly, a higher TFAP2A expression is correlated to a higher expression of the ubiquitin thioesterase enzyme OTUB1 (OTU Deubiquitinase, Ubiquitin Aldehyde Binding 1). The presence of a binding site in the OTUB1 promoter for TFAP2A suggested an enhanced overexpression of OTUB1 when high levels of TFAP2A are detectable in cancer cells.

Of note, molecular docking studies highlighted the formation of hydrogen bonds between solasonine and TFAP2A, with a resulting suppression of its protein levels, lack of binding to the OTUB1 promoter region, and decreased OTUB1 expression. Finally, OTUB1 overexpression favors SLC7A11 deubiquitination, with a resulting higher protein activity and protection from ferroptosis. Overall, solasonine promoted ferroptosis through the TFAP2A/OTUB1/SLC7A11 axis and also decreased the expression of P-gp and MRP1 efflux pumps involved in the onset of multidrug resistance.

### B. Flavonoids

Flavonoids are a rich class of natural compounds characterized by the presence of a structure made of a backbone of 15 carbon atoms contained in a 2-phenylbenzopyranone scaffold. Based on the unsaturation degree of the pyranone ring and the presence of oxygen-containing functional groups, flavonoids are further subclassified into flavones, isoflavones, flavonols, flavanones, flavanols, chalcones, and anthocyanidins. Moreover, the presence of sugars linked to the heterocyclic backbone allows a further classification in free aglycones and glycosides derivatives (Corradini et al., 2011).

Several plants and vegetables are the main source of compounds belonging to this chemical class, which have been deeply investigated especially for their beneficial antioxidant and antitumoral effects (Ullah et al., 2020). As a class of generally safe compounds, research focused on the identification of flavonoids that are capable of modulating the ferroptotic process (Zheng et al., 2021).

#### 1. 4,4′-Dimethoxychalcone

##### i. *In vitro*

Yang et al. (2022a) reported that 4,4′-dimethoxychalcone promotes ferroptosis, in A549 and 786-O cells through the Keap1/NRF2/HO-1 pathway. In particular, 4,4′-dimethoxychalcone triggered Keap1 ubiquitination, consequent NRF2 nuclear translocation, and HO-1 transcription. Moreover, cells treated with 4,4′-dimethoxychalcone were characterized by a reduced enzymatic activity of ferrochelatase, increased expression of genes involved in lipid peroxidation exacerbation (Prostaglandin-Endoperoxide Synthase 2 *PTGS2*, *ACSL4*, *ALOX15*, P450 oxidoreductase *POR*), and increased expression of glutathione specific gamma-glutamylcyclotransferase 1 (*CHAC1*). The latter was responsible for GSH depletion, as the role of GPX4 in GSH homeostasis was ruled out in A549 cells.

#### 2. Amentoflavone

Amentoflavone is a biflavonoid produced by several plant families, including *Selaginellaceae*, *Cupressaceae*, *Euphorbiaceae*, *Podocarpaceae*, and *Calophyllaceae*, and shares the same beneficial effects found in other flavonoids, such as antioxidant, anti-diabetes, and anticancer effects (Yu et al., 2017).

##### i. *In vitro* and *in vivo*

In U251 and U373 human glioma cells, amentoflavone has been shown to induce ferroptosis through an autophagy-dependent mechanism of action (Chen et al., 2020d). More specifically, amentoflavone cell treatment favored AMPK phosphorylation and reduced mTOR and p70S6K phosphorylation. The activation of this pathway suppressed FTH expression by activation of autophagy, as demonstrated also by the increased autophagy-related protein levels of Autophagy Related 5 (ATG5), Autophagy Related 7 (ATG7), Beclin1, and LC3BII. FTH deficiency impaired iron homeostasis and promoted ROS accumulation, mitochondrial damage, lipid peroxidation, and GSH consumption.

#### 3. Baicalin

##### i. *In vitro* and *in vivo*

Kong et al. (2021) demonstrated that baicalin, a flavone found in *Scutellaria baicalensis*, is capable of apoptosis and ferroptosis induction in bladder cancer cells (5637 and KU-19-19) both *in vitro* and *in vivo*. The mechanism of action involves again ROS accumulation and mitochondrial damage. Further, cells treated with baicalin increased TF expression and reduced HO-1 and FTH1. A deeper investigation about the role of FTH1 was performed through cell plasmid transfection with the *FTH1* gene. Overexpression of FTH1 caused reduction of TF expression, reduced iron cell intake, decreased ROS production and consequent ferroptosis mitigation. Therefore, the authors suggested that FTH1 plays a crucial role in ferroptosis induction in bladder cancer.

#### 4. Ginkgetin

##### i. *In vitro* and *in vivo*

Ginkgetin is a biflavonoid obtained from *Gingko biloba* leaves with neuroprotective, antioxidant, antibacterial, and anticancer properties (Adnan et al., 2020).

Its role in ferroptosis has been recently investigated in EGFR wild-type NSCLC (A549, NCI-H460, and SPC-A-1) cell lines in combination with cisplatin, and confirmed by the use of DFO and the improved Fer-1 analog UAMC 3203 (Lou et al., 2021). The combination of cisplatin and ginkgetin was effective in increasing the cytotoxicity toward the tested cell lines when compared with the cytotoxic effects exerted singularly by the two compounds.

In addition, the co-administration enhanced lipid peroxidation, whereas the protein levels of GPX4 and SLC7A11 were decreased. Further, SLC40A1 and TF mRNA expression and protein content were also increased. Finally, the addition of ginkgetin to cisplatin-treated cells blocked the NRF2/HO-1 pathway, with a consequent reduced nuclear translocation of HO-1 and inactivation of NRF2 downstream genes involved in cell survival. Ginkgetin alone did not change the mRNA level of SLC40A1. However, SLC40A1 mRNA levels were increased after combination with cisplatin. It is reported that cisplatin can modulate iron homeostasis (Brown et al., 2020; Lou et al., 2021), so it is reasonable to suppose that it is a cisplatin-related effect that is disentangled from the ferroptosis mechanism probably activated by ginkgetin.

#### 5. Icariside II and luteolin

##### i. *In vitro* and *in vivo*

In renal cell carcinoma, icariside II and luteolin triggered ferroptosis, modulating two different pathways. The former compound exhibited antiproliferative and anti-migration effects by an increase of Fe^2+^, MDA, and ROS levels accompanied by a reduction of GSH levels and p53-independent downregulation of GPX4. Noticeably, icariside II also enhanced the upregulation of miR-324-3p, which, in turn, inhibits the transcription of *GPX4* (Yu et al., 2022). These findings suggested that icariside II could be of particular utility for anticancer treatments regardless of the involvement of p53 in tumor proliferation and invasion.

GSH depletion, ROS accumulation, Fe^2+^ intracellular increased concentration, and disruption of the mitochondrial membrane potential were also observed for luteolin; however, ferroptosis induction was mainly ascribed by an excessive upregulation of HO-1 expression and accumulation of LIP (Han et al., 2022b).

#### 6. Nobiletin

##### i. *In vitro*

Very recently, Feng et al. (2022) described ferroptosis induction in SK-MEL-28 melanoma cells exerted by nobiletin, a polymethoxyflavone found in plants of the *Citrus* genus. Together with the observation of the hallmarks of ferroptosis, the molecule exerted its antitumoral effects through the overexpression of GSK3β, an event that inhibited the activation of the Keap1/NRF2/HO-1 pathway and therefore abrogated this antioxidant defense system. The role of GSK3β in nobiletin-induced ferroptosis was further validated by genetic knockdown or cell transfection with the expression plasmid for GSK3β. Silencing of GSK3β determined the attenuation of lipid peroxidation, decrease of MDA, iron, and ROS levels, and increase of GSH, whereas opposite results were observed after GSK3β overexpression.

### C. Phenols and polyphenols

Phenols and polyphenols are a class of natural compounds characterized by the presence of one or more phenol functional groups that provide strong antioxidant and anticancer effects (Russo et al., 2017; Singla et al., 2019). Flavonoids can be considered as a sub-category of this general class of compounds. Plants, fruits, and vegetables represent the main source of these molecules whose healthy effects are widely exploited in the cosmeceutical and nutraceutical fields (Anunciato and da Rocha Filho, 2012). Induction of ferroptosis has been described also for some compounds belonging to this chemical class.

#### 1. 6-Gingerol

##### i. *In vitro*

6-Gingerol is a phenol found in *Zingiber officinale* with proven anti-inflammatory and anticancer properties (Wang et al., 2014). Its role in ferroptosis modulation was assessed in prostate cancer and lung adenocarcinoma. Prostate cancer cell lines (DU145, PC3, LNCaP) treated with 6-gingerol showed a marked reduction of cell viability and colony formation. Cell migration and invasion have been shown to be less effective after 6-gingerol treatment, and these results were also confirmed after cell stimulation with lipopolysaccharide (LPS).

Considering that LPS plays a role in tumor invasion and aggressiveness, this observation led to the investigation of the expression of protein implicated in the epithelial–mesenchymal transition process. Results showed that 6-gingerol was effective in significantly upregulating the protein levels of E-cadherin and ZO-1 (Zonula occludens-1, also known as Tight junction protein-1). On the other hand, N-cadherin and Vimentin were downregulated, but not in DU145 cells. As far as ferroptosis induction is concerned, 6-gingerol increased ROS levels and it decreased GSH and GPX4 expression levels.

The activation of ferroptotic cell death was confirmed by Fer-1 treatment, which was able to restore cell viability and GSH levels. Of note, NRF2 expression after cell treatment showed a different trend between androgen-dependent and -independent prostate cancer cell lines. Indeed, DU145 and PC3 cells increased NRF2 expression following 6-gingerol, whereas in LNCaP cells NRF2 expression was decreased (Liu et al., 2022a). Tsai et al. (2020) demonstrated that 6-gingerol is a promising anticancer agent also in lung cancer as shown *in vitro* in A549 cells and *in vivo* in tumor-bearing mice through an autophagy-dependent ferroptosis mechanism of action that involves the decrease of the expression of ubiquitin-specific protease 14 (USP14).

Specifically, cell treatment with 6-gingerol was accompanied by the increased expression of proteins responsible for autophagy activation and decreased expression of GPX4, FTH, ATF4, and UPS14. Considering that in normal conditions UPS14 is responsible for the deubiquitination of the K63 site of Beclin-1, the reduced expression of UPS14 after cell treatment with 6-gingerol promotes an inhibition of Beclin-1 deubiquitination with consequent activation of the autophagic pathway.

#### 2. Curcumin

##### i. *In vitro*

Curcumin is a diarylheptanoid curcuminoid whose main biological source is represented by the rhizoma of turmeric. Great attention was placed on this natural compound, considering that several studies demonstrated its strong anticancer, antibiotic, anti-inflammatory, antioxidant, and anti-aging properties (Kotha and Luthria, 2019). The role of curcumin in ferroptosis induction was shown to be contradictory. For instance, in diabetic cardiomyopathy and renal cell damage, curcumin alleviates ferroptosis (Guerrero-Hue et al., 2019; Wei et al., 2022b), whereas in cancer cells it possesses antitumorigenic properties (Costantino et al., 2022).

As well as 6-gingerol, A549 and H1299 cells treatment with curcumin triggered ferroptosis through an increase of Beclin-1 and LC3 levels and a reduction of p62 levels, with consequent formation of autolysosomes and autophagy. Further, GPX4, SLC7A11, and GSH levels were decreased, whereas iron, ROS, and peroxidized lipids levels were significantly increased (Tang et al., 2021c). In MCF-7 and MDA-MB-453 BC cell lines, curcumin dose-dependently reduced cell viability and this result was further magnified when cells were co-treated with erastin. A transcriptomic analysis on MCF-7 and MDA-MB-231 cell lines highlighted that GPX4 is not the most important regulator of ferroptosis induction promoted by curcumin treatment (Li et al., 2020c).

Indeed, HO-1 seems to play the most predominant role, as curcumin has been reported to be a strong HO-1 inducer (Balogun et al., 2003). These findings are in agreement with the results obtained by our research group in TNBC cells (Consoli et al., 2022). Specifically, the best results in terms of reduction of cell viability and HO-1 induction in the MB-MDA-231 cell line were obtained with a 30 μ*M* concentration of curcumin. The same concentration was also capable of evoking an increase in Fe^2+^, ROS and lipid peroxide levels, decrease GSH cell content and mitochondria membrane potential, but not GPX4 levels.

##### ii. *In vivo*

Ferroptotic cell death was showed to be dependent on the ability of curcumin to increase both SLC1A5 mRNA and protein levels, followed by increased glutamine cell intake and consequent lipid peroxidation and ROS production also *in vivo* (Cao et al., 2022; Gao et al., 2015; Luo et al., 2018). Moreover, a recent study (Zhang et al., 2022c) reported curcumin volatile component curcumenol effect on a lung cancer xenograft model. The compound was able to induce ferroptosis through activation of lncRNA H19/miR-19b-3p/FTH1 axis, leading to the reduction of GPX4, FTH1, and SLC7A11 levels and increase of HO-1 expression. Tumor volume was significantly reduced after curcumenol treatment (200 mg/kg), and the effect was reversed by DFO co-administration.

#### 3. Erianin

##### i. *In vitro* and *in vivo*

Erianin is a bisbenzyl phenol compound isolated from *Eria coronaria* and *Dendrobium chrysotoxum* with ascertained antitumoral, antiangiogenic, and antimetastatic effects (Zhang et al., 2019b). The viability of KU-19-19 and RT-4 bladder cancer cells was reduced in a dose-dependent and time-dependent manner after treatment with erianin, with cell cycle arrest at the G2/M phase. Ferroptosis induction in the same cell lines was evidenced by decreased GSH, FTH1, HO-1, glutaminase and xCT/SLC7A11 production and increased ROS, MDA, and iron levels.

An in-depth investigation of the mechanisms involved in ferroptosis induction highlighted that erianin inhibited NRF2 activation. Cells treatment with an NRF2 activator hampered ferroptosis induction, whereas NRF2 knockdown confirmed its pro-ferroptotic role in bladder cancer cell death. Similar results were also obtained *in vivo*, where erianin reduced tumor growth in a xenograft model and was well tolerated (Xiang et al., 2021).

A different study focused on erainin effects on H460 and H1299 lung cancer cell lines (Chen et al., 2020c). Ferroptosis induction was assessed both *in vitro* and *in vivo*, with results almost superimposable to those previously described in bladder cancer. In addition, the authors of this work speculated on erianin interaction and activation of the Ca^2+^/calmodulin signaling pathway. Calmodulin is a Ca^2+^-regulating protein that modulates the activity of the L-type voltage-dependent Ca^2+^ channel (LVDCC), which can also participate in Fe^2+^ ion intake, intracellular accumulation, and consequent ROS production, an event that culminates in ferroptosis induction.

#### 4. Honokiol

##### i. *In vitro*

The antitumoral properties of honokiol, a lignan isolated from Magnolia species (Rauf et al., 2021), have been poorly investigated in the context of ferroptosis. Lai et al. (2022) recently evaluated its antitumoral properties in a panel of acute myeloid leukemia (AML) cell lines (THP-1, U-937, and SKM-1). In the same manner as the activity of curcumin in BC, results showed that heme oxygenase-1 encoding gene (HMOX-1) upregulation is the event that drives ferroptosis following honokiol cell treatment.

This observation was confirmed by the protective effects obtained with cells co-treatment with honokiol and an HO-1 inhibitor (zinc protoporphyirin, ZnPP). Further, honokiol also upregulated SAT1, a protein engaged in polyamine catabolism whose activation is regulated by p53. SAT1 induction mutually raises the expression levels of ALOX15, which, in turn, promotes lipid peroxidation (Ou et al., 2016). Finally, leukemia cells treated with honokiol overexpressed the SLC7A11 protein, and this event could be seen as a contradiction since a reduced expression of this protein correlates to ferroptosis induction.

However, the authors of this work proposed that the observed overexpression could be seen as a compensatory mechanism aimed at restoring the redox homeostasis impaired by HMOX-1 upregulation. Nevertheless, SLC7A11 upregulation also favors the intracellular cystine import, an event that can culminate in cell toxicity (Koppula et al., 2021).

### D. Saponins

Saponins are a class of natural biomolecules with high structural complexity, remarkable foaming properties in an aqueous environment (Man et al., 2010), and countless pharmacological effects (Juang and Liang, 2020). They possess a tetracyclic core (aglycone) linked to linear or branched oligosaccharides through glycosidic bonds. On the basis of the chemical structure of the aglycone backbone, saponins are subclassified into triterpenoid or steroidal saponins (Sparg et al., 2004). To the latter subgroup belong some compounds whose anticancer effects have been recently explained by their ability to promote ferroptosis in different cancer cell lines.

#### 1. Formosanin C

##### i. *In vitro*

Formosanin C is a diosgenin saponin found in the leaves of *Paris chinensis* with confirmed anticancer properties in colorectal, hepatic, ovarian, and lung cancer (Cui et al., 2019; Lee et al., 2009; Li et al., 2014; Yang et al., 2015). Results obtained with BC cell lines displayed that formosanin C induced ferroptosis activation, with better results obtained in MDA-MB-231 cells when compared with luminal A MCF-7 cells. Moreover, formosanin C performed better than cisplatin in inhibiting MDA-MB-231 cell growth and also increased MDA-MB-231 cisplatin sensitivity. Ferroptosis was demonstrated by lipid peroxidation, GPX4 depletion, increased TfR1 and FTH1 expression, and downregulation of xCT expression.

Further, formosanin C additionally triggered cell death by ferritinophagy, as demonstrated by downregulation of ferroportin, upregulation of the autophagy marker LC3-II/LC3-I, and autophagosome formation (Chen et al., 2022a). Formosanin C-dependent ferroptosis and autophagy were also observed in HCC cells (HepG2 and Hep3B); however, different results have been obtained between the two cell lines. Indeed, differences in their proteome may be accountable for the higher susceptibility of HepG2 cells toward ferroptosis.

Differently from Hep3B, HepG2 cells are p53 wild-type and this event is strictly correlated to the lower expression of SLC7A11 protein (Jiang et al., 2015). In addition, HepG2 cells express high levels of p62, a protein that competes with Keap1 for the binding to NRF2 (Sun et al., 2016b). Formosanin C cell treatment determined a reduction of the expression of p62, which could decrease NRF2 overactivation and protection from a ferroptotic cell death mechanism.

Above all, differently to Hep3B cells, HepG2 cells are also characterized by lower FTH1 and higher NCOA4 levels that are required for FT degradation and ferritinophagy. Therefore, HepG2 cells are basally more sensitive to autophagy, and formosanin C cell treatment enhanced this form of cell death that was coupled to ferroptosis induction (Lin et al., 2020).

#### 2. Ophiopogonin B

##### i. *In vitro* and *in vivo*

Very recently, Li et al. (2022c) conducted a bioinformatic analysis aimed at identifying ferroptosis-related genes and prognosis in NSCLC patients. The study brought to light a 12-gene signature where *AURKA*, the Aurora kinase A encoding gene, was reported to act as a negative regulator of ferroptosis. The high expression of *AURKA* in A549 cells led to the choice of this cell line for biological studies. Ophiopogonin B, a saponin found in *Ophiopogon japonicum* with reported antitumoral properties, was effective in inducing ferroptosis through *AURKA* downregulation. Cell treatment with ophiopogonin B determined a reduced expression of FTH1, FTL, and *AURKA*, downregulation of GPX4, reduced levels of MMP, GSH depletion, increased expression of ACSL4, PTGS2, SLC7A5, and PHKG2 (Phosphorylase Kinase Catalytic Subunit Gamma 2), increased levels of MDA and intracellular iron. Accordingly, similar results were obtained following *AURKA* silencing, whereas its plasmid-mediated overexpression showed opposite effects.

To further validate the experimental data, cells overexpressing *AURKA* were treated with ophiopogonin B. Results showed that *AURKA* overexpression partially counteracted the effects previously observed after saponin administration, confirming that AURKA acts as an inhibitor of ferroptosis in A549 cells. Finally, the antiproliferative effects exerted by ophiopogonin B were also substantiated *in vivo*.

### E. Terpenes and terpenoids

Terpenes represent a class of compounds produced by plants, fungi, and algae through the mevalonate or methylerythritol phosphate pathways (Tholl, 2015). Their chemical classification in sesquiterpenes, diterpenes, and triterpenes depends on the number of repetitions of the isoprene monomer. As one of the widest and most important classes of natural compounds, terpenes found several applications in pharmaceutics and cosmetics (Jaeger and Cuny, 2016).

Of note, several reports assessed the anticancer properties of natural and semi-synthetic derivatives belonging to this chemical class (Islam, 2017; Ren and Kinghorn, 2019; Yang and Dou, 2010). As a consequence, the antitumoral properties of some terpenes in the context of ferroptosis induction have been taken into consideration and intriguing results have been obtained in several cancer cell lines, when such compounds have been used in stand-alone or combination treatments.

#### 1. Carnosic acid

##### i. *In vitro*

In oral squamous cell carcinoma, the benzenediol diterpene carnosic acid, a natural compound extracted from *Rosmarinus officinalis* and *Salvia officinalis*, reversed cisplatin resistance in CAL27 and SCC9 cells and promoted ferroptosis through the increase of ROS and lipid peroxidation levels and decrease of GSH. Ferroptosis induction was mediated by the ability of carnosic acid to decrease the overactivation of the NRF2/HO-1 axis and downregulating xCT in cisplatin-resistant cells. The involvement of NRF2 in cisplatin resistance and ferroptosis inhibition was also confirmed in transfected cells overexpressing NRF2, HO-1, and xCT. Indeed, results showed that the reactivation of this biochemical pathway significantly decreased the effects of carnosic acid and increased cell viability and cisplatin resistance (Han et al., 2022a).

#### 2. Dihydrotanshinone I and tanshinone 2A

##### i. *In vitro*

Dihydrotanshinone I and tanshinone 2A are two diterpene quinones found in *Salvia miltiorrhiza Bunge*, also known as Danshen, a Chinese herb endowed with antitumoral properties. The antiproliferative effects of these phytochemicals find their rationale in the promotion of ferroptosis in breast and GC, respectively. MCF-7 and MDA-MB-231 cell treatment with 10 μ*M* dihydrotanshinone I was more effective than gemcitabine, 5-fluorouracil, and oxaliplatin in terms of growth inhibition. The same concentration was able to increase the MDA level in both cell lines and to decrease both GPX4 activity and expression.

##### ii. *In vivo*

Studies performed in mice xenografted with MCF-7 cells demonstrated that dihydrotanshinone I reduced the tumor volume of about 70% in only 2 weeks with no significant side effects (Lin et al., 2019). BGC-823 and NCI-H87 GC cells treated with tanshinone 2A underwent ferroptosis through upregulation of the p53 protein, consequent downregulation of SLC7A11, and reduced xCT expression. Moreover, tanshinone 2A also caused PTGS2 upregulation, CHAC1 increased expression, ROS production, lipid peroxidation, and GSH depletion. Ferroptosis induction was also observed *in vivo* in a BGC-823 cells xenograft model, with results superimposable to those observed *in vitro* (Guan et al., 2020).

#### 3. Kayadiol

##### i. *In vitro*

He et al. (2022) recently reported for the first time that kayadiol, a diterpenoid isolated from the japanese conifer *Torreya nucifera*, exerts ferroptosis in extranodal natural killer/T cell lymphoma (NKTLC). This molecule was selectively cytotoxic for YT cells, and the ferroptotic mechanism of action seemed to be related to the involvement of p53 as previously described for tanshinone 2A. This hypothesis was confirmed after p53 knockdown, an event that restored cell viability through the reactivation of the SLC7A11/GPX4 axis. Further, cells co-treatment with kayadiol and L-asparaginase or cisplatin showed a strong antitumoral synergistic effect.

#### 4. Oridonin and ponicidin

##### i. *In vitro*

Oridonin and ponicidin are two *ent*-kaurane tetracyclic diterpenoids characterized by the presence of an α,β-unsaturated ketone moiety. This functional group is responsible for the cytotoxicity exerted by this class of compounds. Indeed, it has been reported that the α,β-unsaturated moiety can bind to free thiols, cysteine, or GSH through a Michael addition, leading to ROS generation and inactivation of molecules and enzymes whose free thiol groups are necessary for their biological activity, such as GPX4, thioredoxin (Trx), and peroxiredoxin (Sun et al., 2021).

Xu et al. reported that oridonin and ponicidin, two ent-kaurane terpenoids from *Rabdosia rubescens*, caused ferroptosis on TE1 esophageal cancer cells and SW1990 pancreatic cancer cells, respectively (Cui et al., 2022; Zhang et al., 2021a). Cell treatment with oridonin or ponicidin determined a reduction in their GSH/GSSG ratio and increase of their Fe^2+^, ROS, and MDA intracellular levels. Moreover, the GPX4 activity and cysteine intracellular content were both decreased. On SW1990 cells, ponicidin administration significantly altered the mitochondrial membrane potential and reduced the mRNA levels of SLC3A2 and SLC7A11.

Finally, these studies demonstrated that ferroptosis was caused by the imbalance of the γ-glutamyl cycle. In TE1 cells, Gamma-Glutamyltransferase 1 (GGT1) and GCLC activities were decreased as well as the intracellular levels of glutamate. Considering that the latter is necessary for the synthesis of GSH, a deficiency of the activity of this biochemical pathway can lead to an imbalanced intracellular redox status and consequent oxidative stress and ferroptosis induction.

#### 5. Pseudolaric acid B

##### i. *In vitro* and *in vivo*

*Pseudolarix kaempferi* root bark represents the main source of pseudolaric acid B, a tricyclic diterpene that demonstrated ferroptosis induction in glioma through multiple mechanisms of action (Wang et al., 2018). Rat C6 and human SHG-44, U87 and U251 glioma cells were treated with the compound and showed a significant inhibition of cell viability in a dose- and time-dependent fashion, with iron accumulation representing the driving force that caused ferroptosis. The increased iron intracellular accumulation and consequent ROS production and lipid peroxidation were caused by overexpression of the TF receptor, after pseudolaric acid B administration, whereas the possible downregulation of FHT and ferroportin was excluded. Contrariwise, their levels were increased after cell treatment, maybe as a cytoprotective mechanism against iron overload.

Moreover, pseudolaric acid also increased the activity and the protein levels of NOX4, an NOX enzyme responsible for H_2_O_2_ production. Finally, the diterpene object of this study also upregulated p53, which, in turn, downregulated xCT and enhanced GSH and cysteine depletion.

#### 6. Artesunate and dihydroartemisinin

##### i. *In vitro* and *in vivo*

Among the natural compounds investigated for ferroptosis induction, the most numerous and important results have been obtained from artemisinine derivatives. Artemisinin, the main product isolated from *Artemisia annua*, is a sesquiterpene lactone endoperoxide endowed with anti-malarial properties that showed also interesting immunosuppressive and anticancer effects (Efferth and Oesch, 2021; Haynes, 2001; Kiani et al., 2020; Martino et al., 2019; Slezakova and Ruda-Kucerova, 2017). Artemisinin derivatives, namely artesunate and dihydroartemisinin, showed interesting antitumoral effects in multiple cancer cell lines by induction of apoptosis, autophagy, and ferroptosis, and they proved to be suitable compounds for the attenuation of multidrug resistance through co-administration with other anticancer drugs (Du et al., 2019; Markowitsch et al., 2020; Vakhrusheva et al., 2022).

In U251 glioblastoma cells, artesunate augmented the expression levels of Divalent Metal (Ion) Transporter 1 (DMT1), TfR, NCOA4, and FT, decreased the expression of FPN1 and GPX4, and additionally activated ferroptosis through the MAPK/ERK and MAPK/p38 pathways as a consequence of the increased ROS content (Song et al., 2022). Accordingly, it was observed that ERK, p38, and JNK can be recruited following erastin treatment and activate MAPK pathway (Cullinan and Diehl, 2006; Gao et al., 2018; Xie et al., 2016). Similarly, dihydroartemisinin increased TfR expression and downregulated GPX4 in U87 and A172 glioblastoma cell lines, although the expression levels of other proteins involved in the ferroptotic process were not affected. The only exception was seen with HO-1, whose upregulation determined a higher intracellular iron content that was responsible for cell death (Yi et al., 2020).

In liver cancer cells, dihydroartemisinin triggers ferroptosis by activation of the unfolded-protein response mediated by CHAC1 increased transcription. Notably, ferroptosis was demonstrated to be not dependent on the p53 cell status as wild-type, mutated, and null p53 cells all underwent cell death after dihydroartemisinin treatment (Wang et al., 2021b). In addition, in Huh7 cells co-treated with low doses of sorafenib and artesunate, cell death was more pronounced when compared with sorafenib or artesunate monotherapy, and ferroptosis was observed through the activation of ferritinophagy both *in vitro* and *in vivo* (Li et al., 2021).

CHAC1 activation was also responsible for the artesunate-inducible cell death of Burkitt's lymphoma DAUDI and CA-46 cells (Wang et al., 2019b), whereas in U2932 and SU-DHL4 diffuse large B cell lymphoma cell lines, apoptosis, ferroptosis, and autophagy induction were promoted by inhibiting the STAT3 signaling pathway (Chen et al., 2021b). In HL-60, THP-1, and KG1 AML cell lines, dihydroartemisinin induced cell cycle arrest at the G0/G1 phase after treatment for 12 h, mitochondrial dysfunction, and downregulation of the mTOR/p70S6k signaling pathway, leading to an autophagy-dependent degradation of FT, which contributed to ferroptosis together with GPX4 degradation and decreased GSH level (Du et al., 2019).

In the multidrug-resistant K562/adriamycin (ADM) leukemia cell line, dihydroartemisinin reversed ferroptosis resistance by modulation of the expression levels of GPX4 and proteins involved in iron homeostasis and contributed to cell resensitization toward ADM (Zhang et al., 2022d). Artemisinin derivatives also displayed interesting antiproliferative properties in lung cancer cells (Zhang et al., 2021c). Of note, Yuan et al. reported dihydroartemisinin ability to reduce mRNA and protein content of the DNA Primase Subunit 2 (PRIM2), an enzyme required for DNA damage repair, in XWLC-05 and NCI-H23 lung cancer cells.

Moreover, dihydroartemisinin-dependent PRIM2 decreased expression was associated with downregulation of SLC7A11, lipid membrane damage, and GSH depletion (Yuan et al., 2020). One of the main issues encountered in tumor eradication is also represented by the interaction of tumor cells with other cellular populations that reside in the tumor microenvironment. Interestingly, dihydroartemisinin proved to be efficacious in remodeling the phenotype of lung cancer TAMs from a pro-tumor M2 phenotype to an antitumor M1 phenotype by induction of ferroptosis (Li et al., 2022d). Specifically, dihydroartemisinin-dependent GPX4 decreased levels and TfR-1 upregulation favored lipid peroxidation and ROS accumulation, consequently determining DNA damage and activation of nuclear factor kappa-light-chain-enhancer of activated B cells (NF-κB).

The new pro-inflammatory intracellular environment established by ferroptosis induction allowed an M2 to M1 phenotype shift, with promising antitumoral effects observed when TAMs were co-cultured with Lewis lung cancer cells (LLC). Likewise, remodulation of immune cells was observed *in vivo* in an orthotopic PDAC tumor model. In particular, Panc02 cells treated with dihydroartemisinin showed a p53/ALOX12-dependent activation of ferroptosis coupled with reduction of M2 and MDSCs populations and expansion of CD8^+^ T, NK, and NKT cells that could contribute to an enhanced antitumor immunity (Zhang et al., 2022a).

#### 7. Caryophyllene oxide

##### i. *In vitro*

Caryophyllene oxide is an anti-inflammatory, antioxidant, and antitumoral bicyclic sesquiterpenoid epoxide derivative of β-caryophyllene, with main natural sources represented by basil (*Ocimum* spp.), salvia (*Salvia glutinosa*), and *Syzygium cordatum* (Fidyt et al., 2016). In HCC (HCCLM3 and HUH7 cells), caryophyllene oxide demonstrated antiproliferative properties thanks to its ability to induce ferritinophagy both *in vitro* and *in vivo* (Xiu et al., 2022).

Indeed, cell treatment with caryophyllene oxide dose-dependently raised up ROS and Fe^2+^ content leading to MDA increased production as a result of lipid peroxidation. Simultaneously, GSH levels were reduced, as well as the expression levels of NRF2, FTH1, HO-1, NQO1, and GPX4. On the other hand, caryophyllene oxide increased the expression levels of NCOA4, which, in turn, binds to LC3-PE triggering ferritinophagy.

#### 8. β-Elemene

##### i. *In vitro* and *in vivo*

The sesquiterpene β-elemene, whose natural source is represented by the Chinese herb *Rhizoma curcumae*, reduced KRAS mutant colon cancer cells growth and migration in combination with cetuximab through cell cycle arrest at the G0/G1 phase and ferroptosis induction (Chen et al., 2020b). The wound-healing assay performed on HCT116 and Lovo cells showed that epithelial-mesenchymal transformation was inhibited by downregulation of vimentin, N-cadherin, MMP-9, Slug, and Snail proteins, whereas the levels of E-cadherin were upregulated. Ferroptosis was observed by the identification of its common hallmarks, including reduced expression of glutaminase, GPX4, SLC7A11, SLC40A1, and FTH1. Finally, the cetuximab-β-elemene co-administration did not show significant side effects *in vivo* in an orthotopic murine cancer cell model.

#### 9. Eupaformosanine

##### i. *In vitro* and *in vivo*

Eupaformosanine is a bicyclic germacranolide sesquiterpene found in *Eupatorium cannabinum* characterized by a five-membered ring containing a lactone functional group and an α,β-unsaturated moiety. Very recently, eupaformosanine antiproliferative effects were described in MDA-MB-231 and MDA-MB-468 TNBC cell lines and BALB/c nude mice tumor xenograft (Wei et al., 2022a). The molecule was able to induce cell cycle arrest at the G2/M phase and disrupt the mitochondrial membrane potential.

Ferroptosis involvement as a mechanism of cell death after eupaformosanin treatment was demonstrated by analyzing ROS levels, GPX4 activity, FTH1 expression, and intracellular iron levels. Further, ferroptosis inhibitors were able to rescue cell viability, confirming ferroptosis role in cancer cell death. A nMDA-MB-231 cell line bearing a mutation in TP53 (R280K) was chosen to evaluate the potential role of eupaformosanin in triggering ferroptosis through modulation of p53 activity in light of the observation that multiple mutations in this protein hinder therapeutic efficacy.

Surprisingly, eupaformosanin was shown to promote ferroptosis through mutant p53 ubiquitination. Of note, differences in the modulation of ferroptosis are observed on the basis of a p53 wild-type or mutant phenotype. As a matter of fact, if wild-type p53 causes ferroptosis, mutant p53 inhibits it through the downregulation of SAT1, GLS2 and upregulation of SLC7A11. Interestingly, cell treatment with eupaformosanin increased GLS2 and SAT1 expression, with the latter increasing the expression of ALOX15, whereas SLC7A11 levels were decreased, thereafter reducing the activity of the xCT system. The role of mutant p53 in ferroptosis inhibition was further confirmed after its knockdown, which brought to cell viability rescue and opposite results observed with eupaformosanin cell treatment.

#### 10. Eupalinolide B and tagitinin C

##### i. *In vitro* and *in vivo*

Structurally related to eupaformosanin, the natural compounds eupalinolide B and tagitinin C, isolated from *Eupatorium lindleyanum* and *Tithonia diversifolia*, respectively, both induced ferroptosis by modulation of the NRF2/HO-1 axis. In SMMC-7721 and HCCLM3 hepatocarcinoma cell lines, eupalinolide B inhibited cell cycle progression at the S phase by modulation of CDK2 and cyclin E1. Mitochondria morphological changes, reduced expression of GPX4, and ROS production were identified as symptoms of ferroptosis induction.

The intracellular oxidative imbalance promoted by eupalinolide B induced the upregulation of HO-1, which, in turn, caused ER stress. Concurrently, ER stress activated the JNK signaling pathway and inhibited cell migration. However, ferroptosis inhibition with DFO or Fer-1 did not abolish the blocking of cell migration, whereas cell treatment with the ER stress inhibitor 4-phenylbutyric acid or with the ROS scavenger N-acetyl-L-cysteine reversed the anti-migration effects of eupalinolide B.

Therefore, in HCC, ferroptosis and cell migration inhibition caused by eupalinolide B seem to be two antiproliferative mechanisms not intertwined with each other (Zhang et al., 2022e). In HCT116 colon cancer cells, tagitinin C did not induce ferroptosis through downregulation of GPX4, considering that after cell treatment, no significant changes in its expression levels were observed. Instead, cytochrome POR levels were upregulated, causing oxidation of cell membrane PLs. Concurrently, tagitinin C induced ER stress through the PERK/NRF2/HO-1 pathway.

##### ii. *In vitro*

Finally, tagitinin C increased the susceptibility of HCT116 cells to ferroptosis in combination with erastin or RSL3, providing interesting insights for new potential therapeutic treatments (Wei et al., 2021).

#### 11. Glycyrrhetinic acid and oleanolic acid

##### i. *In vitro*

Among the triterpene subclass, the *Glycyrrhiza glabra* derived glycyrrhetinic acid exerted interesting cytotoxic effects on MDA-MB-231 and BT-549 BC cell lines (Wen et al., 2021). Differently from the other mechanisms of action described so far, glycyrrhetinic acid promoted ferroptosis not only by GSH depletion and SLC7A11 downregulation, but also by upregulation of iNOS and NOX subunit p47^phox^, which executed lipid peroxidation through the production of nitric oxide and peroxinitrite radical species. Further, cells treated with glycyrrhetinic acid did not show any relevant changes in GPX4 expression levels, whereas its protein activity was decreased.

##### ii. *In vitro* and *in vivo*

Structurally related to glycyrrhetinic acid, the pentacyclic compound oleaonolic acid successfully induced ferroptosis in cervical cancer (Xiaofei et al., 2021). Oleanolic acid activated the ACSL4 pathway leading to GPX4 and GSH decreased levels, overexpression of TfR1, reduced FTH1 levels, and ROS overproduction.

#### 12. Poricoic acid A

##### i. *In vitro* and *in vivo*

The mushroom *Wolfiporia extensa*, also known as Poria cocos, is a traditional Chinese medicine used for multiple therapeutic applications (Li et al., 2019a). This mushroom is the main source of the triterpene poricoic acid A, whose pro-ferroptotic and autophagy inducing properties in T-cell acute lymphoblastic leukemia have been recently reported (Chen et al., 2022b). MOLT-3, ALL-SIL, Jurkat, and RPMI-8402 cancer cell lines treated with poricoic acid A underwent cell cycle arrest at the G2/M phase, showing that one of the cell death mechanisms caused by the natural compound was apoptosis.

Increased ROS content, GSH loss, FTH, and GPX4 decreased expression, MDA increased levels, and mitochondrial dysfunction also suggested the involvement of ferroptosis, as demonstrated by increased cell viability after cell treatment with Fer-1. Further, modulation of the AMPK/mTOR/S6 pathway caused a higher production of the Beclin-1, LC3BII, and ATG5 proteins, which contributed to autophagic cell death.

It should be noted that effects of AMPK activation on ferroptosis are heavily context dependent. Several studies also report an inhibitory effect of AMPK caused by inhibition of acetyl-coenzyme A (CoA) carboxylase (ACC), which is necessary for the synthesis of PUFA, substrates for lipid peroxidation. However, AMPK-driven suppression of PUFA synthesis is likely not relevant for ferroptosis suppression in cells with low basal ACC activity. On the contrary, other studies report a pro-ferroptotic effect of AMPK, as in the case of beclin 1, which binds to SLC7A11 to mediate ferroptosis induction in an AMPK-dependent manner (Lee et al., 2020; Li et al., 2020a; Yan et al., 2023).

#### 13. Ursolic acid

##### i. *In vitro* and *in vivo*

Ursolic acid mechanism of cell death in several tumor cell lines was observed to not be dependent from autophagy, but apoptosis and ferroptosis (Li et al., 2022b). The combination of ursolic acid and sorafenib in Hep3B and BEL-7402 human hepatoma cells, H1299, A-427, and SK-LU-1 lung cancer cells, T47D and MCF-7 human BC cells, LoVo and HCT116 human colon cancer cells, AGS and SCG7901 human GC cells, and human PC3 prostate cancer cells resulted in a drastic decrease in cell viability compared with single monotherapy, whereas no cytotoxicity was observed in healthy cells.

*In vivo* experiments with an HCT116 tumor xenograft model showed that the combination therapy was effective in reducing tumor growth and weight with no substantial side effects. The pro-ferroptotic mechanism was investigated and seemed to not be related to changes in the expression of FTH, ACSL4, NRF2, and SLC3A2, but to the decreased protein levels of SLC7A11 and GPX4 activity.

Of note, unlike ursolic acid, it was shown that treatment with ER stress inhibitor tauroursodeoxycholic acid (TUDCA) or calcium chelator 1,2-Bis (2-aminophenoxy) ethane-N,N,N′,N′-tetraacetic acid tetrakis (acetoxymethyl ester) (BAPTA-AM) leads to lower accumulation of the core components of ESCRT-III machinery in the plasma membrane, dampening ferroptosis (Dai et al., 2020; Tang et al., 2021b).

Tables 1–7 summarize all the compounds discussed in this review to simplify the identification of common mechanisms of action between the different chemical classes.

**Table 1.** Alkaloids

Compound	Natural source	*In vitro *studies			*In vivo *studies	Mechanism of ferroptosis induction	References
		Cell lines	Concentration	Inhibitors of ferroptosis			
Capsaicin 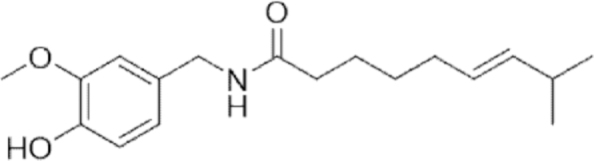	*Capsicum*	A549, NCI-H23	200, 100 μ*M*	Fer-1	None	Decreased activity of the SLC7A11/GPX4 pathway	Liu et al. (2022c)
		U87-MG, U251	121.6, 188.5, 237.2 μ*M*	None	None	ACSL4, 5-HETE, TOS, MDA increased levels, LDH increased activity; GPX4 decreased activity, TAS, and GSH decreased levels	Hacioglu and Kar (2023)
Lycorine 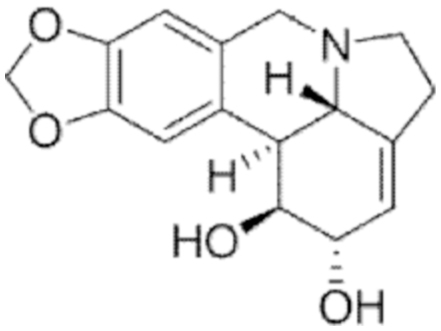	Amaryllidaceae	Caki-1, A498, 786-O	10 μ*M*	Fer-1	None	ACSL4, 5-HETE, 12-HETE, 15-HETE, and MDA increased levels; GPX4 decreased activity, reduced GSH/GSSG ratio	Roy et al. (2018)
Piperlongumine 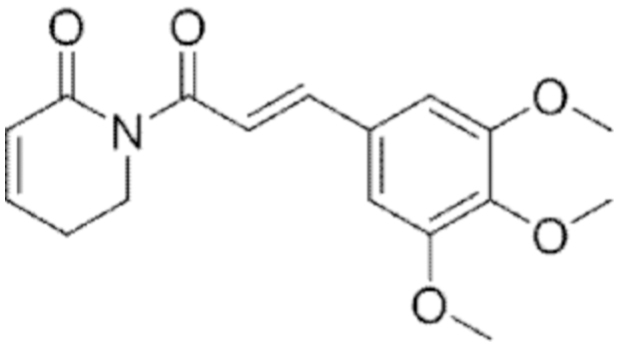	*Piper longum*	MIAPaCa-2, PANC-1	From 4 to 14 μ*M*	Fer-1, Lip-1, DFO	None	Increased ROS content and mRNA HO-1 levels	Yang et al. (2022c)
Sanguinarine 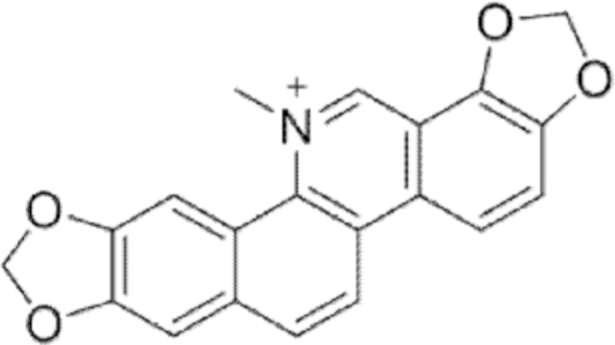	*Sanguinaria canadensis*	HeLa	3 μ*M*	Fer-1, Trolox, DFO	None	H_2_O_2_ production, increased Fe^2+^ and LPO contents, decreased GSH and SLC7A11 levels	Alakkal et al. (2022)
Solasonine 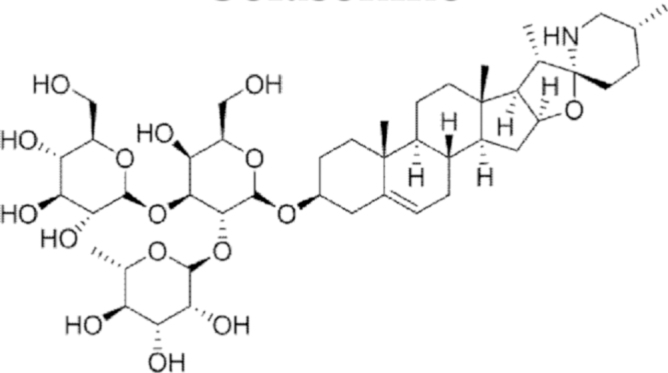	Solanaceae	Calu-1, A549	20, 30 μ*M*	Fer-1, DFO	None	ROS production, increased Fe^2+^ and LPO levels, mitochondiral membrane depolarization, decreased GSH, cysteine, and SLC7A11 levels	Zeng et al. (2022)
		HepG2, HepRG	15 μg/mL	Fer-1, DFO	Hepatic cancer xenograft model (BALB/c nude mice)	ROS production and disruption of the activity of the GSH redox system	Jin et al. (2020)
		PANC-1, CFPAC-1	From 5 to 50 μ*M*	Fer-1	Pancreatic cancer mice xenograft models	Antimetastatic effects through blockage of the TFAP2A/OTUB1/SLC7A11 pathway; reduced expression of efflux pumps (P-gp and MRP1)	Liang et al. (2022)

5-HETE, 5-hydroxyeicosatetraenoic acid; ACSL4, acyl-CoA synthetase long chain family member 4; DFO, deferoxamine; Fer-1, ferrostatin-1; GPX4, glutathione peroxidase 4; GSH, glutathione; GSSG, GSH disulfide; HeLa, human cervical cancer; HO-1, heme oxygenase 1; LDH, lactate dehydrogenase; Lip-1, liproxstatin-1; LPO, lipid peroxidation; MDA, malondialdehyde; mRNA, messenger RNA; MRP1, multidrug resistance protein 1; OTUB1, out Deubiquitinase, Ubiquitin Aldehyde Binding 1; ROS, reactive oxygen species; SLC7A11, solute carrier family 7 member 11; TAS, total antioxidant status; TFAP2A, transcription factor activating enhancer binding protein 2 alpha; TOS, total oxidant status.

**Table 2.** Flavonoids

Compound	Natural source	*In vitro *studies			*In vivo *studies	Mechanism of ferroptosis induction	References
		Cell lines	Concentration	Inhibitors of ferroptosis			
4,4′-Dimethoxychalcone 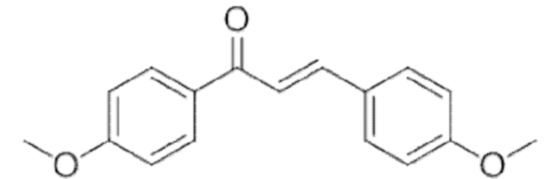	*Angelica keiskei* *Koidzumi*	A549, 786-O	50, 100 μ*M*	DFO	None	Keap1 ubiquitination, NRF2/HO-1 axis activation, reduction of ferrochetalase activity, increased expression of *PTGS2*, *ACSL4*, *ALOX15*, *POR*, and *CHAC1*	Yang et al. (2022a)
Amentoflavone 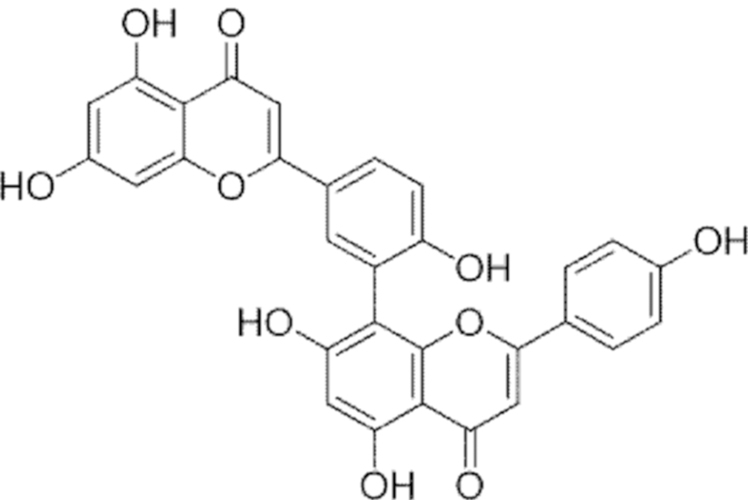	Selaginellaceae, Cupressaceae, Euphorbiaceae, Podocarpaceae, Calophyllaceae	U251, U373	10, 20 μ*M*	Fer-1, DFO	Glioblastoma xenograft murine model (BALB/c nude mice)	Activation of the AMPK/mTOR/p70S6K pathway, FTH decreased expression, increased levels of autophagy-related proteins, ROS production, LPO, GSH depletion, and mitochondrial damage	Chen et al. (2020d)
Baicalin 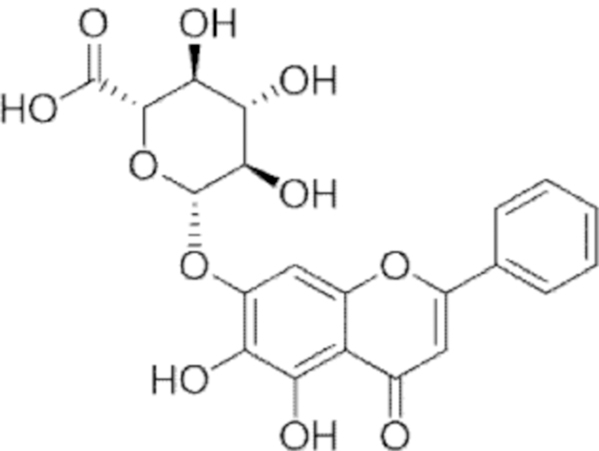	*Scutellaria Baicalensis*	5637, KU-19-19	50, 60 μg/mL	DFO	Bladder cancer xenograft murine model (BALB/c nude mice)	Increased transferrin expression, reduced HO-1 and FTH1 levels	Kong et al. (2021)
Ginkgetin 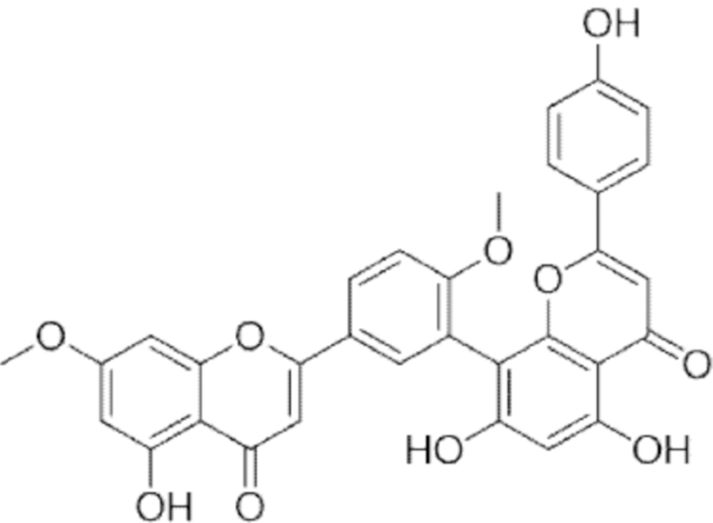	*Gingko biloba*	A549, NCI-H460, SPC-A-1	5 μ*M*	DFO, UAMC 3203	NSCLC xenograft nude mice model	In combination with cisplatin: reduced GPX4 and SLC7A11 levels, increased content of SLC40A1 and transferrin, inhibition of NRF2/HO-1 pathway	Lou et al. (2021)
Icariside II 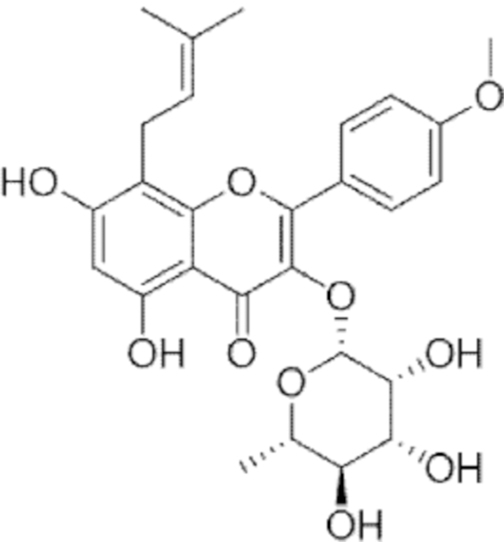	*Epimedium* *Koreanum*	ACHN, A498, 786-O, Caki-1	10, 20, 40 μ*M*	Fer-1, Lip-1, DFO	Renal cell (ACHN and Caki-1 cells) carcinoma xenograft model (BALB/c nude mice)	p53-independent downregulation of GPX4, upregulation of miR-324-3p	Yu et al. (2022)
Luteolin 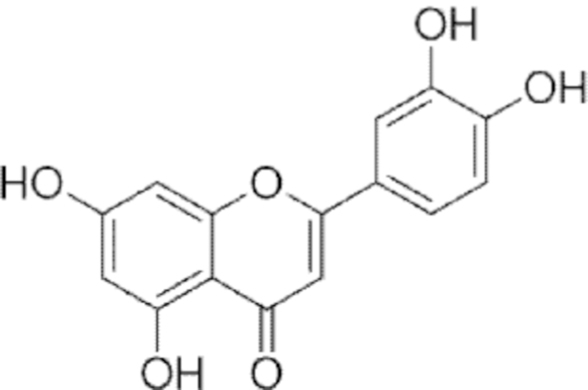	Ginkgoaceae, Anacardiaceae, Apiaceae, Asteraceae, Cucurbitaceae, Euphorbiaceae, Fabaceae, Lamiaceae	786-O, OS-RC-2	30 μ*M*	Fer-1, deferiprone (DFP)	Renal cancer xenograft model (BALB/c nude mice)	HO-1 upregulation, GSH loss, ROS production, increased Fe^2+^ levels, and alteration of the mitochondrial membrane potential	Han et al. (2022b)
Nobiletin 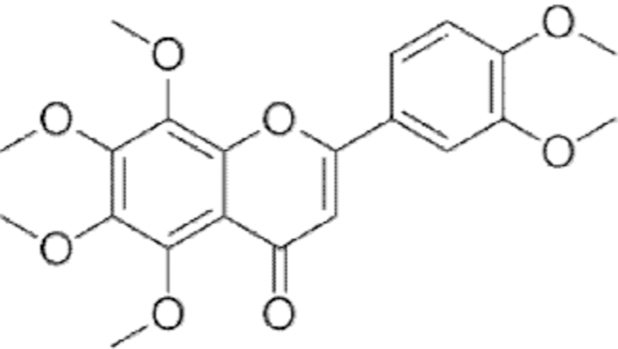	*Citrus*	SK-MEL-28	15 μ*M*	Fer-1, Lip-1	None	GSK3β overexpression, inhibition of the Keap1/NRF2/HO-1 axis	Feng et al. (2022)

ALOX, lipoxygenase; *CHAC1*, gamma-glutamylcyclotransferase 1; FTH1, ferritin heavy chain 1; GSK3β, Glycogen Synthase Kinase 3 Beta; Keap1, kelch-like ECH-associated protein 1; NRF2, nuclear factor erythroid 2 (NF-E2)-related factor 2; NSCLC, non-small-cell lung cancer; p53, tumor protein p53; POR, P450 oxidoreductase; PTGS2, Prostaglandin-Endoperoxide Synthase 2; SLC40A1, solute carrier family 40 member 1.

**Table 3.** Phenols and Polyphenols

Compound	Natural source	*In vitro *studies			*In vivo *studies	Mechanism of ferroptosis induction	References
		Cell lines	Concentration	Inhibitors of ferroptosis			
6-Gingerol 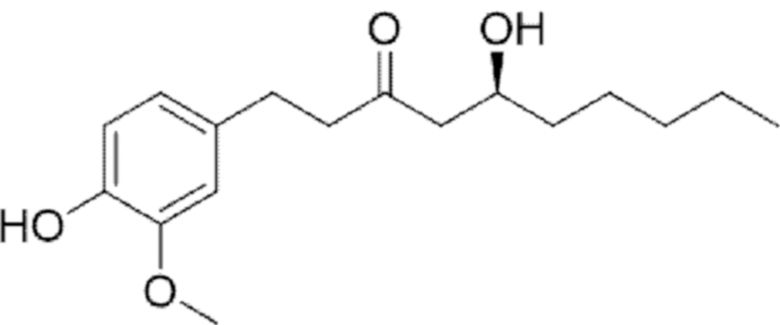	*Zingiber officinale*	DU145, PC3, LNCaP	100 μ*M*	Fer-1	None	ROS accumulation, decreased levels of GSH and GPX4, NRF2 modulation	Liu et al. (2022a)
		A549	20, 40, 80 μ*M*	None	Lung cancer xenograft model (BALB/c nude mice)	USP14, GPX4, FTH, ATF4 decreased expression, and autophagy activation	Tsai et al. (2020)
Curcumin 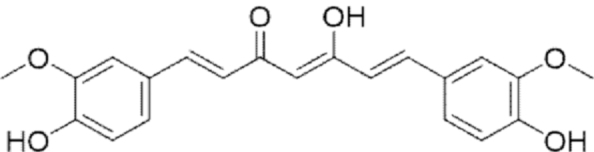	*Curcuma longa*	A549, H1299	30 μ*M*	Fer-1	LLC-bearing mice	Increased expression of autophagic proteins, decreased p62, GPX4, SLC7A11 and GSH levels, LPO, iron, and ROS accumulation	Tang et al. (2021c)
		MCF-7, MDA-MB-453	20 μ*M*	Fer-1, DFO	Breast cancer xenograft model (BALB/c nude mice)	Increased SLC1A5 mRNA and protein levels and glutamine intake, LPO and ROS production	Cao et al. (2022)
		MB-MDA-231	30 μ*M*	Fer-1, DFO, Trolox	None	ROS production, lipid peroxidation, Fe^2+^ increased levels, decreased GSH content, and HO-1 induction	Consoli et al. (2022)
Erianin 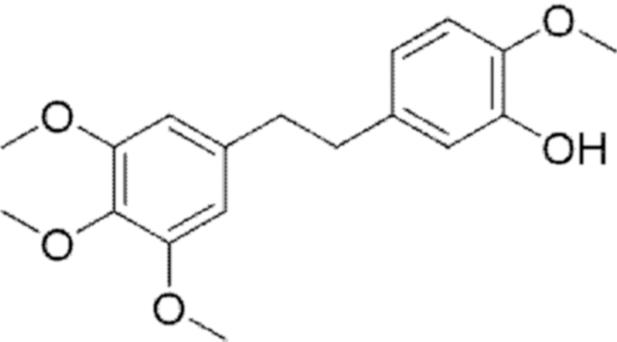	*Eria coronaria, Dendrobium chrysotoxum*	KU-19-19, RT-4	20, 40, 60, 80, 100 μg/mL	DFO	Bladder cancer xenograft tumor model (BALB/c nude mice)	Decreased GSH, FTH1, HO-1, glutaminase, and xCT/SLC7A11, increased ROS, MDA, and iron levels, and NRF2 inhibition	Xiang et al. (2021)
		H460, H1299	50, 100 n*M*	Fer-1, Lip-1	Lung cancer xenograft tumor model (BALB/c nude mice)	Activation of the Ca^2+^/calmodulin signaling pathway	Chen et al. (2020c)
Honokiol 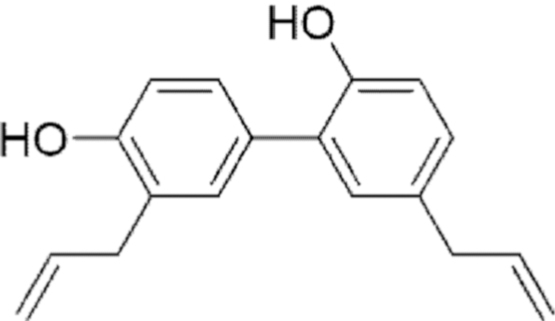	*Magnolia*	THP-1, U-937, SKM-1	20, 30, 35 μ*M*	Fer-1	None	HO-1, SAT1 and ALOX15 upregulation	Lai et al. (2022)

LLC, Lewis lung cancer cells; SAT1, spermidine/spermine *N^1^*-acetyltransferase 1; SLC1A5, solute carrier family 1 member 5; USP14, ubiquitin-specific protease 14.

**Table 4.** Saponins

Compound	Natural source	*In vitro *studies			*In vivo *studies	Mechanism of ferroptosis induction	References
		Cell lines	Concentration	Inhibitors of ferroptosis			
Formosanin C 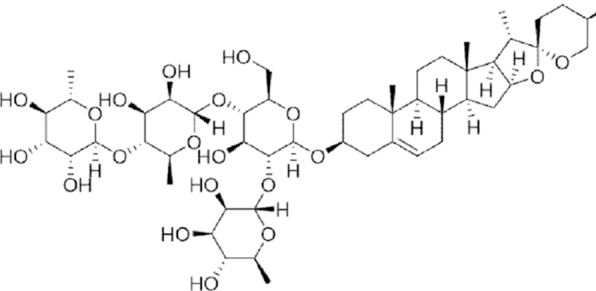	*Paris chinensis*	MDA-MB-231	2, 5, 10 μ*M*	Fer-1, DFO	None	LPO, GPX4 depletion, TfR1 and FTH1 increased expression, xCT downregulation, and ferritinophagy	Chen et al. (2022a)
		HepG2	2.5, 5 μ*M*	Fer-1	None	Reduced p62 expression, inhibition of NRF2 activation, and autophagy	Lin et al. (2020)
Ophiopogonin B 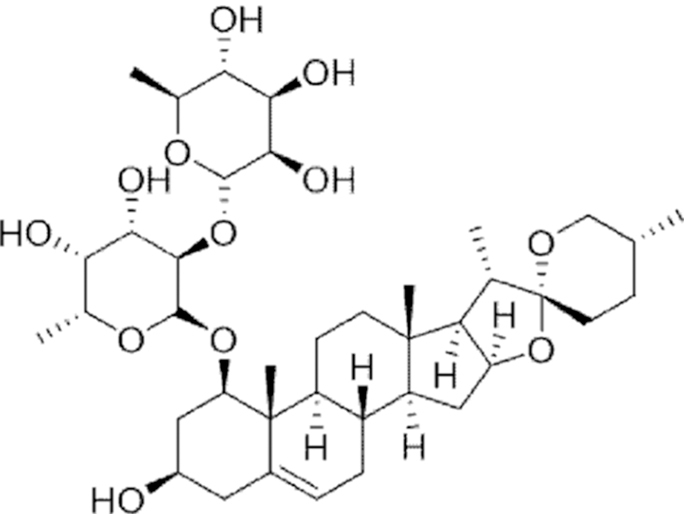	*Ophiopogon japonicum*	A549	2.5, 5 μ*M*	None	A549 orthotopic xenograft lung cancer model (BALB/c nude mice)	Reduced expression of FTH1, FTL, and *AURKA*, reduced levels of MMP, GSH loss, increased expression of ACSL4, *PTGS2*, *SLC7A5*, and PHKG2, increased contents of MDA and Fe^2+^, and downregulation of GPX4	Li et al. (2022c)

*AURKA*, aurora kinase A encoding gene; FTL, ferritin light chain; PHKG2, Phosphorylase Kinase Catalytic Subunit Gamma 2; TfR1, transferrin receptor 1.

**Table 5.** Diterpenes

Compound	Natural source	*In vitro *studies			*In vivo *studies	Mechanism of ferroptosis induction	References
		Cell lines	Concentration	Inhibitors of ferroptosis			
Carnosic acid 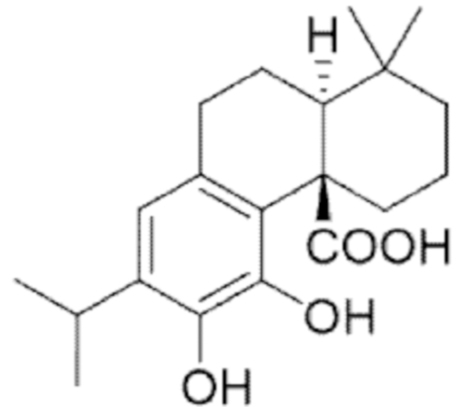	*Rosmarinus officinalis, Salvia Officinalis*	CAL27, SCC9	20 μ*M*	Lip-1	None	Inhibition of NRF2/HO-1 axis, downregulation of xCT	Han et al. (2022a)
Dihydrotanshinone I 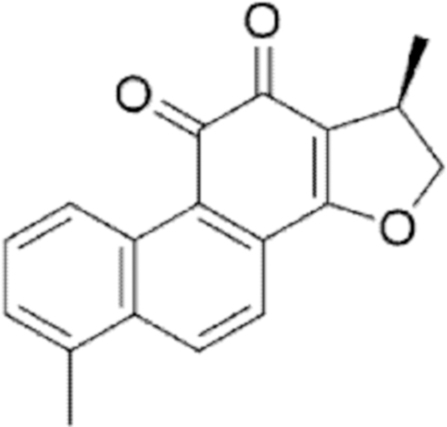	*Salvia miltiorrhiza*	MCF-7, MDA-MB-231	5, 10 μ*M*	None	Breast cancer xenograft model (BALB/c nude mice)	MDA increased levels, decreased GPX4 expression and activity, and GSH depletion	Lin et al. (2019)
Kayadiol 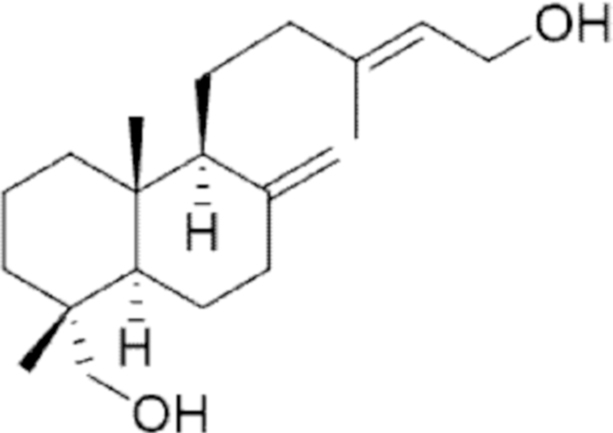	*Torreya nucifera*	YT	6.25, 12.5, 25 μ*M*	Fer-1	None	p53 upregulation, inhibition of the SLC7A11/GPX4 axis	He et al. (2022)
Oridonin 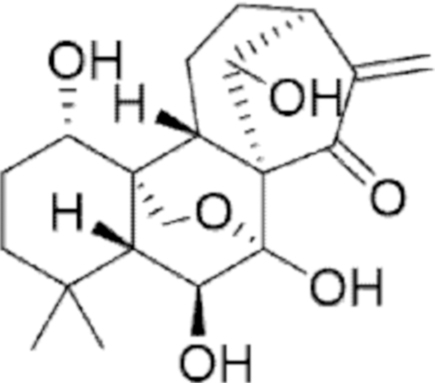	*Rabdosia rubescens*	TE1	15, 20, 30 μ*M*	DFO	None	Reduction of GSH/GSSG ratio, increased Fe^2+^, ROS and MDA concentrations, and GPX4 and Cys reduced levels	Zhang et al. (2021a)
Ponicidin 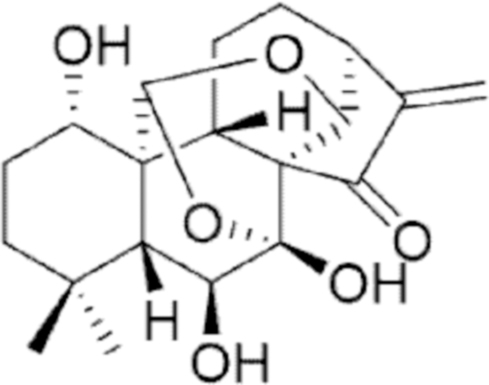	*R. rubescens*	SW1990	10, 20, 30 μ*M*	Fer-1, DFO	None	Alteration of the MMP, reduction of SLC3A2 and SLC7A11 mRNA levels, disruption of the γ-glutamyl cycle	Cui et al. (2022)
Pseudolaric acid B 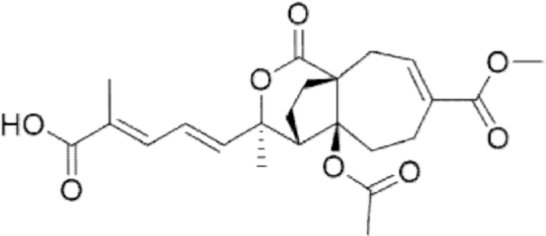	*Pseudolarix kaempferi*	C6, SHG-44, U87, U251	0.5, 2 μ*M*	Fer-1, DFO, Lip-1	Glioma xenograft model (BALB/c athymic nude mice)	Fe^2+^ and ROS accumulation, LPO, TfR1 overexpression, p53 and NOX4 overexpression, GSH depletion, and xCT downregulation	Wang et al. (2018)
Tanshinone 2A 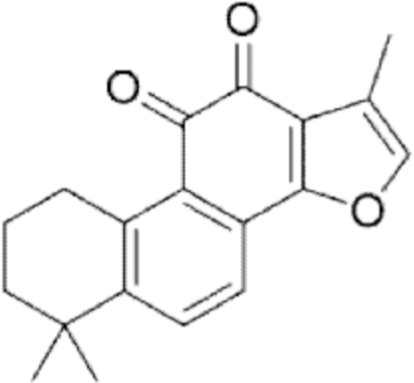	*S. miltiorrhiza*	BGC-823, NCI-H87	2, 4 μ*M*	Fer-1	Gastric cancer xenograft model (NOD CB17-Prkdcscid/NcrCrl mice)	p53 and PTGS2 upregulation, CHAC1 increased expression, SLC7A11 downregulation, and reduced xCT expression	Guan et al. (2020)

Cys, cysteine; NADPH, reduced nicotinamide adenine dinucleotide phosphate; NOX, NADPH oxidase; SLC3A2, Solute Carrier Family 3 Member 2.

**Table 6.** Sesquiterpenes

Compound	Natural source	*In vitro *studies			*In vivo *studies	Mechanism of ferroptosis induction	References
		Cell lines	Concentration	Inhibitors of ferroptosis			
Artesunate 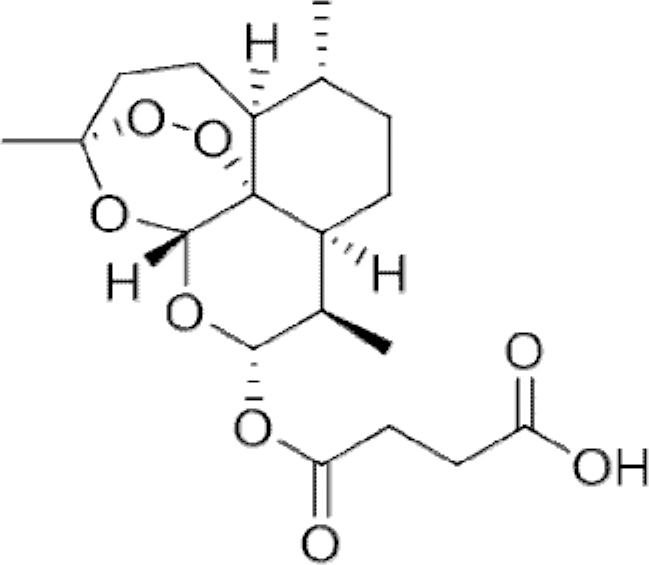	*Artemisia annua*	U251	50, 100 μ*M*	Fer-1	Glioblastoma xenograft model (BALB/c nude mice)	Increased expression of DMT1, TfR, NCOA4, and FTH, decreased expression of FPN1 and GPX4, activation of MAPK/ERK and MAPK/p38 pathways	Song et al. (2022)
		Huh7	200 μ*M*	Lip-1, DFO	Hepatic tumor xenograft model (BALB/c nude mice)	With sorafenib: ferritinophagy	Li et al. (2021)
		DAUDI, CA-46	20 μ*M*	Fer-1, Lip-1, DFO	Lymphoma mouse xenograft model (NOD/SCID mice)	CHAC1 activation	Wang et al. (2019b)
		U2932, SU-DHL4	5, 10, 20 μ*M*	Fer-1	Lymphoma xenograft model (NOD/SCID mice)	Inhibition of the STAT3 signaling pathway	Chen et al. (2021b)
Dihydroartemisinin 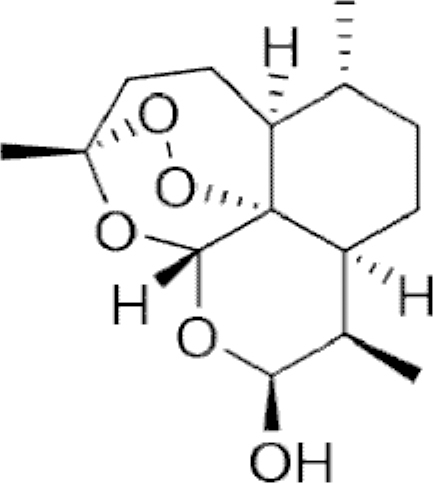	*A. annua*	U87, A172	100 μ*M*	Fer-1	None	Increased TfR expression, HO-1 upregulation, and GPX4 downregulation	Yi et al. (2020)
		PLC/PRF/5, Hep3B, Huh7, HepG2	25, 30, 35 and 40 μ*M*, respectively	Fer-1, DFO	Hepatic tumor xenograft model (BALB/c nude mice)	CHAC1 increased transcription and activation of the unfolded-protein response	Wang et al. (2021b)
		HL-60, THP-1, KG1	10 μ*M*	Fer-1, DFO	Leukemia xenograft model (BALB/c nude mice)	Downregulation of the mTOR/p70S6k signaling pathway, GPX4 degradation, and ferritinophagy	Du et al. (2019)
		K562/ADM	25, 50 μ*M*	Fer-1	None	GPX4 reduced activity, disruption of iron homeostasis	Zhang et al. (2022b)
		XWLC-05, NCI-H23	40, 60 μ*M*	Fer-1	Lung tumor xenograft model (female nude mice)	Reduced PRIM2 mRNA and protein levels, SLC7A11 downregulation, GSH loss, LPO	Yuan et al. (2020)
		LLC	30, 60 μg/mL	Fer-1	Female C57BL/6 LLC-bearing mice	Remodeling of TAMs from M2 to M1 phenotype, GPX4 decreased levels, ROS production, LPO, TfR1 upregulation, DNA damage, and NF-κB activation	Li et al. (2022d)
		Panc1, Panc02	50, 100, 150 μ*M*	Fer-1, Lip-1	Orthotopic pancreatic tumor model (C57BL/6 mice)	Activation of the p53/ALOX12 pathway, M2 to M1 TAMs remodeling	Zhang et al. (2022a)
Caryophyllene oxide 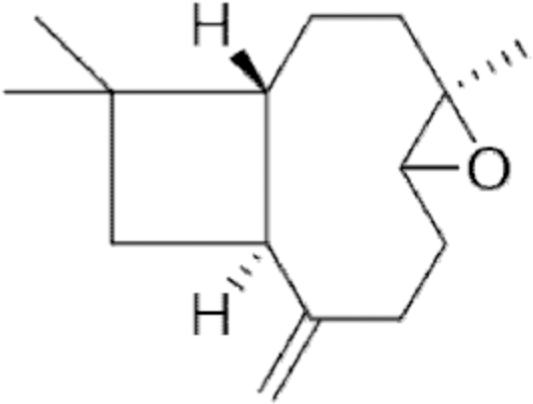	*Ocimum* spp., *Salvia glutinosa, Syzygium cordatum*	HCCLM3, Huh7	40, 80, 160 μ*M*	None	Hepatic tumor xenograft model (BALB/c nude mice)	Ferritinophagy, reduced expression of NRF2, FTH1, HO-1, NQO1 and GPX4, and increased expression of NCOA4	Xiu et al. (2022)
β-elemene 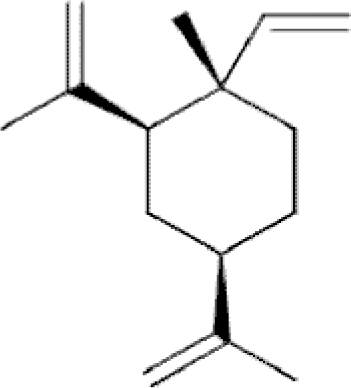	*Rhizoma curcumae*	HCT116, Lovo	125 μg/mL	Fer-1, DFO, Lip-1	Orthotopic murine colon cancer model (BALB/c nude mice)	Reduced expression of glutaminase, GPX4, SLC7A11, SLC40A1, and FTH1	Chen et al. (2020b)
Eupaformosanine 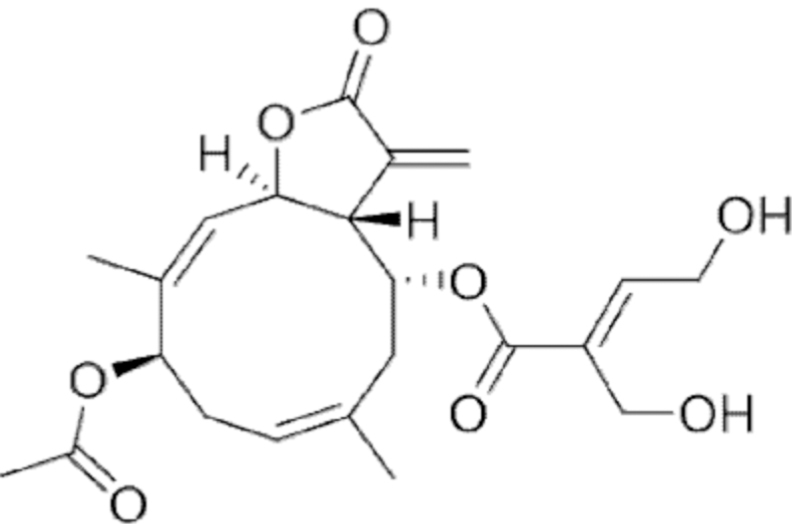	*Eupatorium cannabinum*	MDA-MB-231, MDA-MB-468	4, 8 μ*M*	Fer-1, DFO, Lip-1	Breast cancer xenograft tumor model (BALB/c nude mice)	Ubiquitination of mutant p53, increased GLS2, SAT1, and ALOX15 expression, SLC7A11 reduced levels, and inhibition of xCT activity	Wei et al. (2022a)
Eupalinolide B 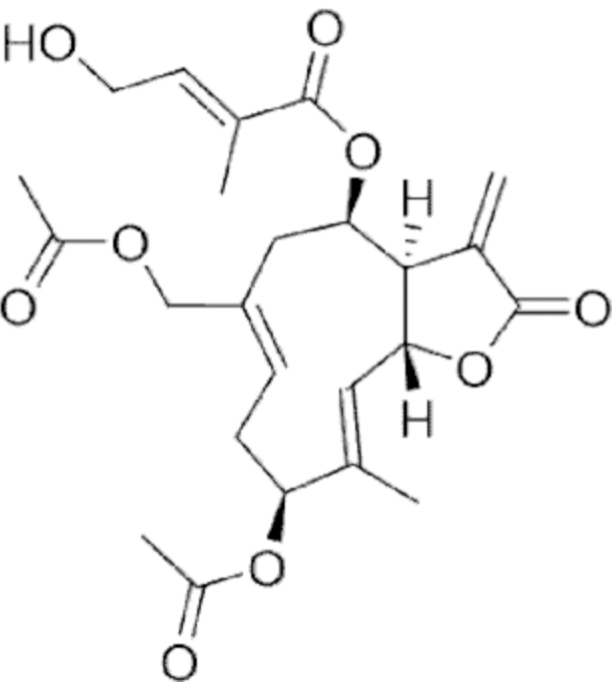	*Eupatorium lindleyanum*	SMMC-7721, HCCLM3	6, 12, 24 μ*M*	Fer-1, DFO	Human hepatic carcinoma xenograft model and PDX model	HO-1 upregulation, ROS production, and GPX4 reduced expression	Zhang et al. (2022c)
Tagitinin C 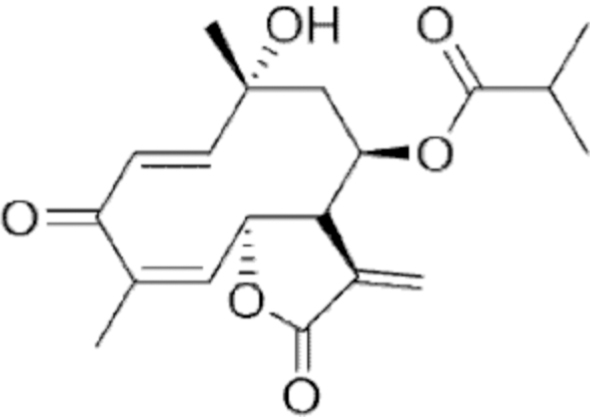	*Tithonia diversifolia*	HCT116	20 μ*M*	Fer-1, DFO	None	POR upregulation, activation of the PERK/NRF2/HO-1 pathway	Wei et al. (2021)

ADM, ÿdriamycin; DMT1, Divalent Metal (Ion) Transporter 1; FPN, ferroportin-1; GLS2, glutaminase 2; NCOA4, nuclear receptor coactivator 4; NF-κB, nuclear factor kappa-light-chain-enhancer of activated B cells; NQO1, NAD(P)H Quinone Dehydrogenase 1; PDX, patient-derived tumor xenograft; PRIM2, primase Subunit 2; TAMs, tumor-associated macrophages.

**Table 7.** Triterpenes

Compound	Natural source	*In vitro *studies			*In vivo *studies	Mechanism of ferroptosis induction	References
		Cell lines	Concentration	Inhibitors of ferroptosis			
Glycyrrhetinic acid 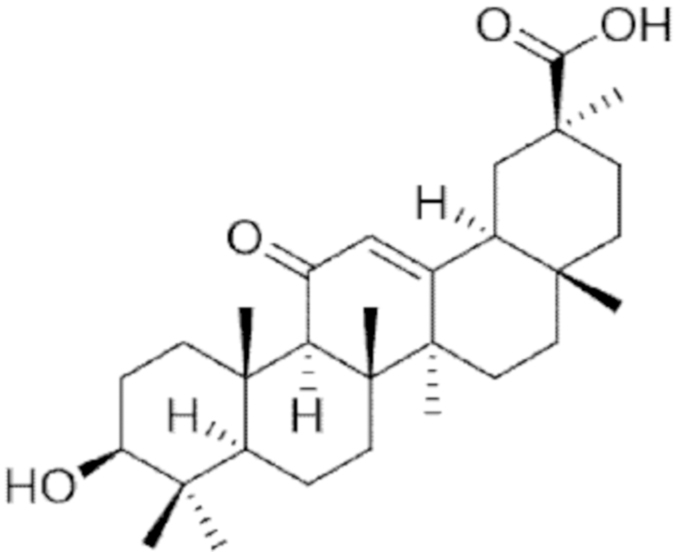	*Glycyrrhiza glabra*	MDA-MB-231, BT-549	40 μ*M*	Fer-1, DFO	None	GSH depletion, SLC7A11 downregulation, and iNOS and NOX subunit p47^phox^ upregulation	Wen et al. (2021)
Oleaonolic acid 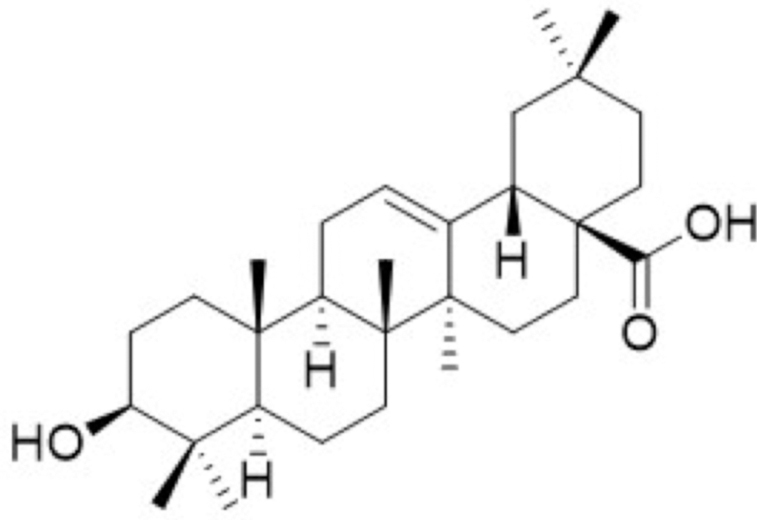	*Olea europaea*, spices and fruits	Hela	5, 10, 20 μ*M*	None	Cervical cancer xenograft model (BALB/c nude mice)	Activation of the ACSL4 pathway, GPX4 and GSH decreased levels, TfR1 overexpression, FTH1 reduced levels, and ROS production	Xiaofei et al. (2021)
Poricoic acid A 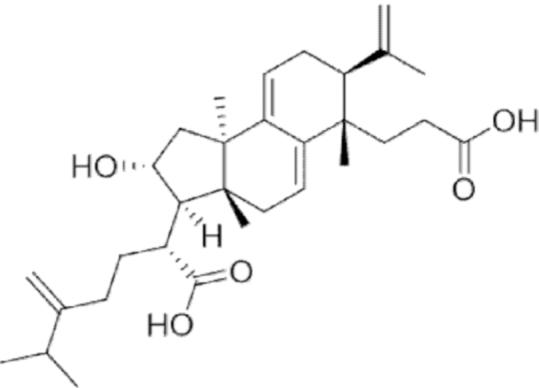	*Wolfiporia extensa*	MOLT-3, ALL-SIL, Jurkat, RPMI-8402	5, 10, 15 μ*M*	Fer-1	Blood cancer xenograft model (BALB/c nude mice)	Activation of the AMPK/mTOR/S6 pathway, ROS production, GSH loss, FTH and GPX4 decreased expression, MDA increased levels, mitochondrial dysfunction, and autophagy	Chen et al. (2022b)
Ursolic acid 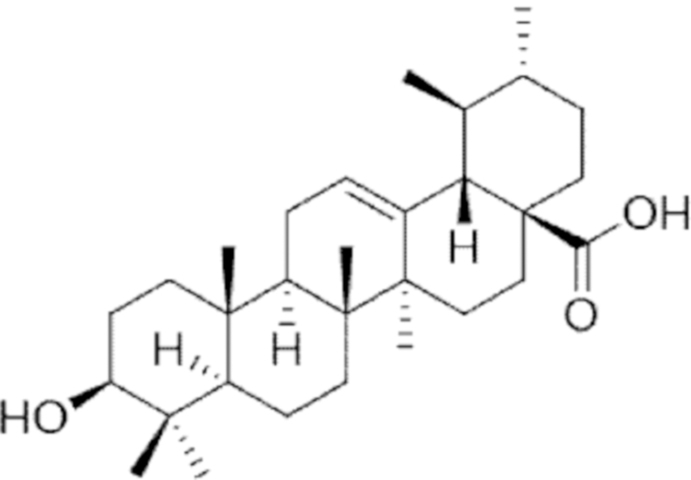	Fruits and spices	Hep3B, BEL-7402, H1299, A-427, SK-LU-1, T47D, MCF-7, LoVo, HCT116, AGS, SCG7901, PC3	6 μ*M*	Fer-1, Lip-1	Colon cancer xenograft model (BALB/c nude mice)	With sorafenib: decreased SLC7A11 levels, inhibition of GPX4 activity	Li et al. (2022b)

## VI. Current Ferroptosis Limitations and Advances Beyond *In Vitro* Research

The possibility of exploiting ferroptosis as novel anticancer therapy is certainly appealing for researchers worldwide; however, a lot has still to be done to unravel and manipulate all the complex mechanisms governing it.

Achieving *in vivo* models of ferroptosis has been a coveted goal not only for cancer related studies but also for neurodegenerative diseases, such as Alzheimer's disease and Parkinson's disease (Ma et al., 2022; Majerníková et al., 2021), ischemia-reperfusion injury (Doll et al., 2017; Du et al., 2023b; Friedmann Angeli et al., 2014), and renal failure (Linkermann et al., 2014; Nehus et al., 2014).

So far, three major functional hallmarks (Dixon et al., 2019) of ferroptosis have been described: (1) It is an iron-dependent cell death. (2) It is driven by lipid peroxidation. (3) It involves defective or inhibited lipid peroxide repair systems such as GPX4.

A combined assessment of all these conditions should be taken into consideration when establishing a ferroptotic model. Moreover, mitigation of cell death by ferroptosis inhibitors (*e.g*., Fer-1, liproxsatin-1) is critical but not a *conditio sine qua non* criteria for ferroptosis validation, especially for *in vivo* models (Devisscher et al., 2018; Ide and Souma, 2022).

Drawbacks of erastin use for *in vivo* models (*e.g*., undesired pharmacological effects) can limit the possibilities of establishing a ferroptosis model as conveniently as for *in vitro* studies; however, to date, different solutions have been proposed: 1. Development of more stable and safe erastin derivatives such as imidazole ketone erastin combined with targeted delivery systems (Larraufie et al., 2015); 2. Depletion of plasma cystine levels by administration of cystIinase, which has been shown to reduce tumor growth *via* ferroptosis, while leaving healthy tissue unharmed (Poursaitidis et al., 2017).

Unconventional hallmarks have been reported for *in vivo* studies, underlining the complexity and heterogeneity of the mechanisms involved in ferroptosis onset and development.

Chen et al. (2022a) recently elucidated the role of Fascin actin-bundling protein 1 (Fascin) in promoting xCT proteasome degradation, modulating cellular vulnerability, and regulating ferroptosis in tamoxifen-resistant cancers. Recently, cytochrome POR has been indicated as a key mediator of ferroptotic cell death, demonstrating to enhance lipid peroxidation independently from the well-established ferroptosis-inducing compound used (Zou et al., 2020). A novel POR regulator, Zoledronic acid, was shown to increase ROS and lipid peroxidation levels in osteosarcoma cells through ferroptosis activation; it was also observed to be able to inhibit osteosarcoma growth *in vivo* using a BALB/c nude mice xenograft model (Jiacong et al., 2023).

Further evidence identifies solute carrier family 25 member 22 (SLC25A22), a mitochondrial glutamate transporter, as a driver of ferroptosis resistance in PDAC. In particular, SLC25A22 acts as a ferroptosis metabolic inhibitor through the synthesis of GSH and MUFAs (Liu et al., 2023).

A common concern regarding the use of ferroptosis-inducing agents for cancer therapy is whether they can be selective toward cancer cells rather than healthy tissue. Data obtained by Wu et al. (2019) both *in vitro* and *in vivo* suggest the potential presence of a dose-responsive window for some cancers that present certain genetic signatures as the ones involved in the cadherin–NF2–Hippo–YAP signaling axis, which is frequently mutated in cancer.

Further, talaroconvolutin A (TalaA) has been proved to be a potential candidate for ferroptosis induction in an *in vivo* model of CRC, showing no evident toxicity in normal tissues as liver and kidney. A pharmacological mechanism of action of TalaA was elucidated, highlighting ALOXE3 and HMOX-1 role in promoting lipid peroxidation (Xia et al., 2020).

The translation of acknowledgment from *in vitro* to *in vivo* studies has also reported exceptionally unexpected results, as discovered by Yang et al. (2023) demonstrating that ferroptosis inhibition sensitizes tumor to bortezomib by increasing misfolded protein-containing exosomes release.

Analyzing ferroptosis from a translational point of view, even if evidence from randomized controlled trials (RCTs) is available to support the clinical use of ferroptosis inhibition in a few scenarios (*e.g*., RCT using N-acetylcysteine in stroke is currently recruiting patients [NCT04918719]), so far most studies have obtained underwhelming results (Devos et al., 2014; Weiland et al., 2019). Concurrently, while growing evidence points to ferroptosis induction as a convincing anti-cancer strategy, clinical outcome data are still missing.

Whereas ferroptosis establishment is relatively straightforward *in vitro*, one of the biggest drawbacks nowadays, which makes translational studies challenging, is the difficulty of accurately determining ferroptosis occurrence *in vivo*, and particularly in humans. Several studies have reported insufficient or not clinically validated outcomes mainly due to small sample size, limiting their statistical power (Wu et al., 2021b). To date, no clinical trial with ferroptosis-associated agents has shown substantial tumor shrinkage or extension of progression-free survival. (Miller et al., 2010; Rybak et al., 2009; Wang et al., 2021a; Wu et al., 2020b).

The concept of cell specificity should be stressed when discussing ferroptosis hallmarks; indeed here, we provide a list of interesting and differentially valuable compounds that can be at least defined as ferroptosis modulators depending on cellular basal conditions and susceptibility, different pathways activation, and/or use of well-established ferroptosis pharmacological inhibitors.

As ferroptosis still lacks a specific, unique, and unequivocal marker useful to identify this particular cell death in a plethora of cancer types and subtypes, every study reported must receive an overall evaluation. This review aims at providing the basis for future studies focusing on novel ferroptotic agents' discovery. Moreover, the use of well-known ferroptosis inhibitors (which can provide a first-line evaluation of ferroptosis onset) has been reported in the tables for each analyzed compound.

## VII. Conclusions and Future Perspectives

Utilization of natural compounds as ferroptosis inducers proved to be a fascinating and promising approach for the treatment of different types of cancer. As phytochemicals generally show a safer pharmacological profile, the potential use of these compounds as coadjuvants for conventional cancer therapies or as stand-alone antineoplastic drugs results in desirable outcomes for the definition of novel therapeutical anticancer protocols.

Taking it into account, cross-referencing of natural compounds databases and ferroptosis-related databases would be of great interest for researchers worldwide to drive and accelerate the discovery of natural compounds with ferroptosis-inducing properties. Exploiting naturally derived compounds can be of use to elucidate novel mechanisms of action implicated in ferroptosis onset and propagation aside the already known mechanism of the synthetic small molecules as erastin or RSL3, as they might interact differently with the molecular targets.

Moreover, the great structural diversity that characterizes natural compounds could be useful for the identification of novel pharmacophores to be used for the synthesis of novel derivatives endowed with better ferroptosis-inducing properties. On the other hand, extraction and recovery processes for these kinds of compounds from their natural source together with their purification procedures can be a limiting factor to be taken into account.

Moreover, it is worth mentioning that for the majority of these compounds the efficacy can be highly dependent on the doses administered, as they both can be beneficial under certain circumstances or detrimental. In 1887, H. Schulz demonstrated for the first time that a toxic substance may induce opposite effects depending on the dose (Arndt-Schulz Law). In recent times, the beneficial effect of exposure to low-grade potentially damaging conditions or very low doses of otherwise toxic compounds has been defined as “preconditioning” and “hormesis.” The term “hormesis” defines adaptive, nonmonotonic, biphasic dose–response relations following an initial disruption in cellular homeostasis (Calabrese and Baldwin, 2002).

Hormesis biological importance has been widely discussed, as it may represent the most acceptable explanation for several occurring phenomena. The main mechanisms involved in hormesis are stress-activated cellular pathways, unfolded protein response, DNA damage response, autophagy, antioxidant system activation, and inflammation, which actually can be also reconducted to ferroptosis regulation (Calabrese et al., 2015; Calabrese et al., 2007; Demirovic and Rattan, 2013; Rattan and Demirovic, 2009).

Notably, although phytochemicals exert beneficial antioxidant activities at low doses, at high doses they can act as pro-oxidants in a wide type of cancer cells by interfering with NRF2 pathway and expression of antioxidant “vitagenes,” such as NQO1, glutathione transferase (GT), GPx, HO-1, sirtuin-1 (Sirt1), and Trx system that are implicated in the dampening of oxidative stress-mediated effects during cancer progression, highlighting the occurrence of the hormesis mechanism (Scuto et al., 2022).

Besides, low availability and pharmacokinetic drawbacks, including poor solubility or high lipophilicity for some particular molecules, could hinder their translation into effective and administrable drugs. Thus, exploiting the advances in drug delivery systems development could be a successful strategy to overcome the difficulties of their vehiculation and to ensure proper targeting. An even more appealing approach can be exploiting nanomaterials as ferroptosis inducers themselves acting as redox modulators through participation in biochemical reactions.

The first nanosystem of this class, the FDA-approved ultra-small poly(ethylene glycol) coated silica nanoparticles that compose Cornel dots (C′ dots), was first reported in 2016 by Kim et al. showing C’ dots ability to adsorb extracellular iron and then release it once entered the cell inducing ROS production, GSH depletion, and ferroptosis onset (Kim et al., 2016).

Considering the lack of a specific target for the majority of the naturally derived compounds, their combination with ferroptosis inducers may have synergistic effects on cell viability potentially working through either ferroptosis and/or other independent pathways. For instance, it has been demonstrated that autophagy plays an important role in ferroptosis modulation by regulating cellular iron homeostasis and ROS generation. Following ferroptosis induction, autophagy is activated and serves as a degrading process of FT (Gao et al., 2016).

Nevertheless, many autophagic markers are implicated in apoptotic cell death, representing a node of crosstalk between these mechanisms. Although autophagy and apoptosis act individually, under specific biological conditions, their crosstalk can synergistically lead to cellular death. Remarkably, nuclear p53 was observed to regulate the transcription of the damage-regulated autophagy modulator (DRAM) that represents another point of crosstalk between autophagy and apoptosis (Chavez-Dominguez et al., 2020).

In conclusion, ferroptosis emerged as an intriguing mechanism of programmed cell death with high aptitude for the eradication of cancerous cells. In this work, we updated the current knowledge on ferroptosis inducers isolated from natural sources with the most valuable and interesting properties. A deeper investigation in this field coupled with a better elucidation of biochemical pathways involved in ferroptosis execution could provide novel insights for this new, complex, yet captivating programmed cell death and help to support future studies.

## Abbreviations Used

σ_1_R: sigma-1 receptor
5-HETE: 5-hydroxyeicosatetraenoic acid
AA: arachidonic acid
ACACA: acetyl-coa carboxylase alpha
ACC: acetyl-coenzyme A (CoA) carboxylase
ACSL3: acyl-CoA synthetase long chain family member 3
ACSL4: acyl-CoA synthetase long chain family member 4
ADM: adriamycin
AGBL2: AGBL carboxypeptidase 2
AKR1C1: aldo-keto reductase family 1 member C1
ALOX: lipoxygenase
ALOXE3: lipoxygenase 3
AML: acute myeloid leukemia
ARE: antioxidant response element
ATG5: autophagy related 5
ATP6V1G2: ATPase H+ transporting V1 subunit G2
*AURKA*: Aurora kinase A encoding gene
BC: breast cancer
BCL2: B-cell lymphoma 2
BH4: tetrahydrobiopterin
BSO: buthionine sulfoximine
C5orf64: lncRNA chromosome 5 putative open reading frame 64
CARS1: cysteinyl-TRNA synthetase 1
CASC1: cancer susceptibility candidate 1
CDKN2A: cyclin dependent kinase inhibitor 2A
CDO1: cysteine dioxygenase 1
ceRNAs: competing endogenous RNAs
CHAC1: gamma-glutamylcyclotransferase 1
CISD1: CDGSH iron sulfur domain 1
CISD2: CDGSH iron sulfur domain 2
CO: carbon monoxide
CoQ10: coenzyme Q10
CRC: colorectal cancer
Cu^2+^: copper
CUL3: cullin3
Cys: cysteine
DFO: deferoxamine
DMT1: divalent metal (ion) transporter 1
DPP4: dipeptidyl-peptidase-4
ECM: extracellular matrix
ELOVL2: elongation of very-long-chain fatty acids-like 2
ER: endoplasmic reticulum
ESCRT-III: endosomal sorting complex required for transport-III
FADS2: fatty acid desaturase 2
FDA: U.S. Food and Drug Administration
FDFT1: farnesyl-diphosphate farnesyltransferase 1
Fer-1: ferrostatin-1
Fe-S: iron-sulfur
FGD3: facio-genital dysplasia 3
FPN: ferroportin-1
FSP1: ferroptosis suppressor protein 1
FT: ferritin
FTH1: ferritin heavy chain 1
FTL: ferritin light chain
G6PD: glucose-6-phosphate dehydrogenase
GC: gastric cancer
GCH1: GTP cyclohydrolase-1
GCL: glutamate-cysteine ligase
GCLC: glutamate-cysteine ligase catalytic subunit
GDF15: growth differentiation factor 15
GLS2: glutaminase 2
GPX4: glutathione peroxidase 4
GR: GSH reductase
GSH: glutathione
GSK3β: glycogen synthase kinase 3 beta
GSS: GSH synthetase
GSSG: GSH disulfide
HAMP: hepcidin antimicrobial peptide
HCCs: hepatocellular carcinomas
HeLa: human cervical cancer
HIC1: hypermethylated in cancer 1
HMOX-1: heme oxygenase-1 encoding gene
HNF4A: hepatocyte nuclear factor 4 alpha
HO: heme oxygenase
HO-1: heme oxygenase 1
HpETE–PEs: hydroperoxy-eicosatetraenoic acid–phosphatidylethanolamines
Keap1: kelch-like ECH-associated protein 1
LDH: lactate dehydrogenase
LINC00968: long intergenic non-protein coding RNA 968
LINC01800: long intergenic non-protein coding RNA 1800
LIP: labile iron pool
Lip-1: liproxstatin-1
LLC: Lewis lung cancer cells
lncRNAs: long noncoding RNAs
LPCAT3: lysophosphatidylcholine acyltransferase 3
LPS: lipopolysaccharide
LSH: lymphoid-specific helicase
LTs: leukotrienes
MARCHF6: membrane associated ring-CH-type finger 6
MDA: malondialdehyde
MDSCs: myeloid-derived suppressor cells
MESH1: human Metazoan SpoT Homolog 1
MGST1: microsomal glutathione S-transferase 1
miRNAs: microRNAs
mRNA: messenger RNA
MRP1: multidrug resistance protein 1
MT-1G: metallothionein-1G
MUFA: monounsaturated fatty acids
MUFA-CoA: MUFA-acyl-CoA esters
NADPH: reduced nicotinamide adenine dinucleotide phosphate
NCOA4: nuclear receptor coactivator 4
ncRNAs: non-coding RNAs
NF-κB: nuclear factor kappa-light-chain-enhancer of activated B cells
NFS1: cysteine desulfurase
NOS2: nitric oxide synthase 2
NOX: NADPH oxidase
NQO1: NAD(P)H quinone dehydrogenase 1
NRF2: nuclear factor erythroid 2 (NF-E2)-related factor 2
NSCLC: non-small-cell lung cancer
OTUB1: OTU deubiquitinase, ubiquitin aldehyde binding 1
p53: tumor protein p53
PCBP1: poly (rC)–binding protein 1
PDAC: pancreatic ductal adenocarcinoma
PDX: patient-derived tumor xenograft
PE: phosphatidylethanolamine
PEBP1: phosphatidylethanolamine-binding protein 1
PHKG2: phosphorylase kinase catalytic subunit gamma 2
PLs: phospholipids
PMN-MDSC: polymorphonuclear myeloid-derived suppressor cell
POR: P450 oxidoreductase
PRIM2: primase subunit 2
PROM2: prominin 2
PTGS2: prostaglandin-endoperoxide synthase 2
PUFA: polyunsaturated fatty acid
PUFA-CoA: PUFA-acyl-CoA esters
PUFA-PL: phospholipid PUFA
PUFA-PLOOH: PUFA-phospholipid hydroperoxide
PVT1: lncRNA plasmacytoma variant translocation 1
RBX1: ring box protein 1
RCTs: randomized controlled trials
ROS: reactive oxygen species
RRM1: ribonucleotide reductase subunit 1
RSL3: RAS-selective lethal molecule 3
SAS: sulfasalazine
SAT1: spermidine/spermine *N^1^*-acetyltransferase 1
SCD1: stearoyl CoA desaturase 1
SEC14L2: SEC14 like lipid binding 2
SERPINA3: serpin family a member 3
SLC1A4: solute carrier family 1 member 4
SLC1A5: solute carrier family 1 member 5
SLC25A22: solute carrier family 25 member 22
SLC39A8: solute carrier family 39 member 8
SLC3A2: solute carrier family 3 member 2
SLC40A1: solute carrier family 40 member 1
SLC7A11: solute carrier family 7 member 11
sMaf: small musculoaponeurotic fibrosarcoma proteins
STEAP3: six-transmembrane epithelial antigen of the prostate 3
SUSD3: sushi domain containing 3
TalaA: talaroconvolutin A
TAMs: tumor-associated macrophages
TAS: total antioxidant status
TF: transferrin
TFAP2A: transcription factor activating enhancer binding protein 2 alpha
TFAP2C: transcription factor AP-2 gamma
TfR1: transferrin receptor 1
TNBC: triple negative breast cancer
TOS: total oxidant status
TPRG1: tumor protein P63 regulated 1
Trx: thioredoxin
TXNRD1: thioredoxine reductase 1
Ub: ubiquitin
USP14: ubiquitin-specific protease 14
ZIP: ZRT/IRT-like protein
